# From Columns to Networks: Search toward the Elusive Single Gyroid with *π*‐Shaped Polyphilic Liquid Crystalline Block Molecules

**DOI:** 10.1002/smsc.202500157

**Published:** 2025-05-22

**Authors:** Silvio Poppe, Changlong Chen, Yu Cao, Feng Liu, Carsten Tschierske

**Affiliations:** ^1^ Institute of Chemistry Martin‐Luther‐University Halle‐Wittenberg Kurt‐Mothes‐Straße 2 06120 Halle Germany; ^2^ Shaanxi International Research Center for Soft Matter State Key Laboratory for Mechanical Behavior of Materials Xi'an Jiaotong University Xi'an 710049 P. R. China

**Keywords:** double gyroids, liquid crystals, network phases, *p*‐terphenyl, reticular self‐assemblies, single diamond networks, tiling patterns

## Abstract

Polyphilic block molecules form a wide range of new liquid crystalline (LC) phases with complex morphologies on a nanometer scale. Herein the soft self‐assembly of *π*‐shaped *p*‐terphenyl‐based bolapolyphiles having two adjacent aliphatic side chains at the central benzene ring (catechol dialkyl ethers) is reported with a focus on the design of single‐network structures. Depending on the length of the side chains and temperature a series of polygonal honeycombs, a zeolite‐like LC, a lamellar phase, and two segmented network phases with cubic symmetry is found. In these networks self‐assembled glycerol spheres, forming the junctions, are interconnected by coaxial *p*‐terphenyl bundles. Upon side‐chain elongation, a double‐gyroid phase with three‐way junctions is replaced by the single diamond having a four‐way junction network. However, the single gyroid supposed to be formed by further side‐chain expansion could not be observed; instead, the LC self‐assembly breaks down completely. It is hypothesized that the formation of single‐network phases by bottom‐up self‐assembly in soft matter systems requires a minimum junction valence of at least 4 to stabilize the networks.

## Graphical abstract

A full spectrum of π‐shaped bolapolyphiles with self‐assembled phases ranging from honeycombs to segmented networks via a zeolite‐like structure is reported. The phase sequence suggests that self‐assembled single‐network phases require junction valence numbers of at least 4 to be thermodynamically stable.

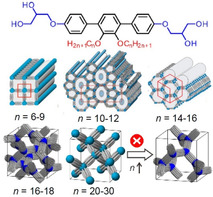

## 1 Introduction

**Figure 1:**
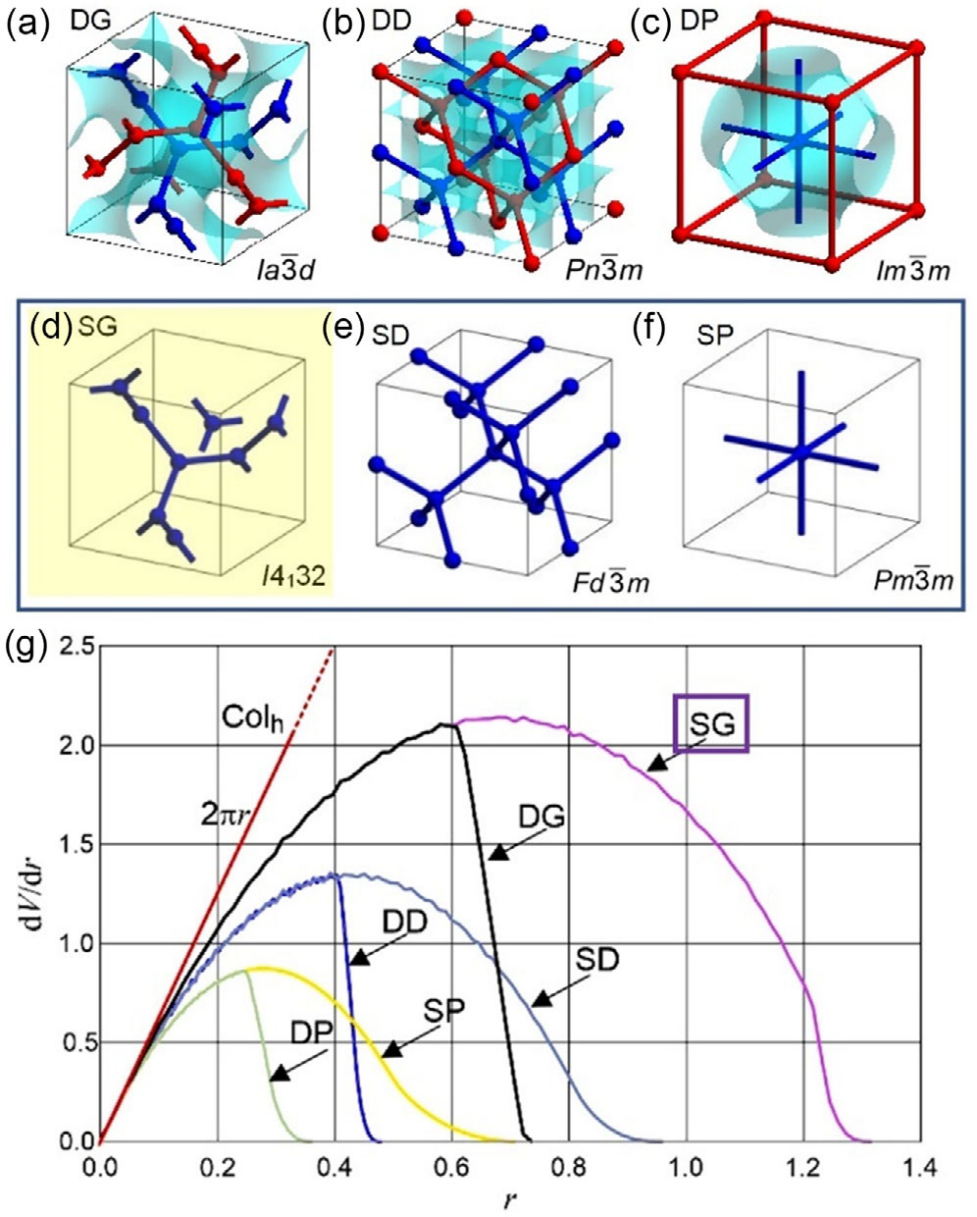
a–c) Double‐network cubic phases with corresponding space groups and their infinite minimal surfaces (blue) and d–f) the corresponding single networks; g) the radial distribution *dV/dr* for these network phases, where *V*(*r*) is that part of the unit cell volume that is within a distance *r* from the closest network segment. The curves show the increase in occupied volume as the radius (*r*) of the cylindrical network segments increases (the lengths of the network segments are for all phases normalized to 1). Reproduced with permission.^[^ ^33^ ^]^ Copyright 2020, American Chemical Society.

**Figure 2:**
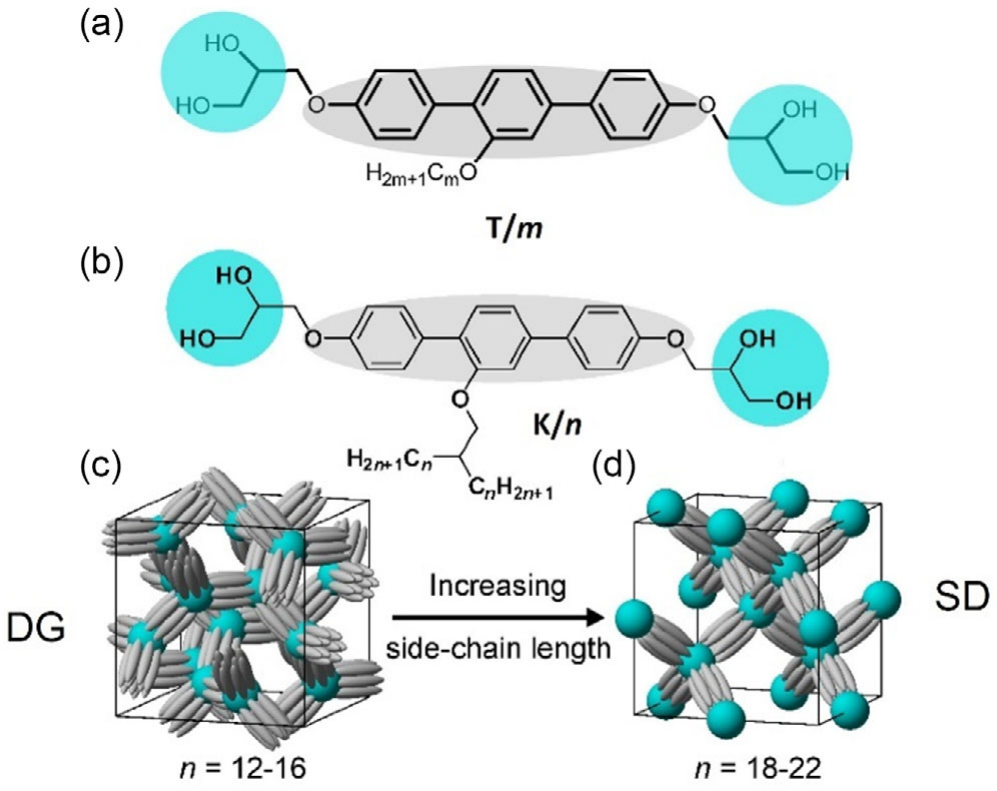
*p*‐Terphenyl‐based BPs: a) **T** **/** ***m*** and^[^ ^75^ ^]^ b) **K** **/** ***n***. c,d) The latter forming the DG and SD segmented network phases, respectively.^[^ ^31^, ^32^ ^]^

The spontaneous development of structural and chemical complexity in fluid systems has enabled the development of life.^[^ ^1^, ^2^ ^]^ The periodic biophotonic nanostructures of beetles, butterfly wing sales, and birds’ feathers are examples for such spontaneously developed complex functional structures, in this case providing iridescent structural colors.^[^ ^3^ ^]^ The morphologies of these nanostructures span almost the complete amphiphilic phase range from lamellar via columnar to spherical modes of self‐assembly, including networks.^[^ ^4^, ^5^ ^]^ There are three common types of networks with cubic space groups, the gyroid (G), diamond (D), and primitive (P) with three‐, four‐ or six‐way junctions, respectively. Each of them can consist of a single network (single gyroid, SG, single diamond, SD, single primitive, SP, with growing junction valence, see **Figure** **1**d–f) or as double networks composed of two mutually interwoven networks (double gyroid, DG, double diamond, DD, double primitive, DP, also known as Plumber's Nightmare, see Figure 1a–c). The double‐network structures are well known and commonly found in amphiphilic systems like lyotropics,^[^ ^6^, ^7^ ^]^ solvent‐free amphiphilic liquid crystals (LCs),^[^ ^8^, ^9^, ^10^ ^]^ and block copolymers,^[^ ^11^ ^]^ with the DG being the most common in all cases.^[^ ^4^, ^6^, ^7^, ^12^, ^13^ ^]^ In contrast, the single network counterparts are rare and do not spontaneously form in a bottom‐up self‐assembly process, though simulations predict their existence for special block copolymer architectures.^[^ ^14^, ^15^, ^16^, ^17^, ^18^, ^19^ ^]^ Their production requires top‐down approaches, post‐modification techniques like shifting or removing one network from the alternating DG (DG^A^) or interference with kinetic or surface effects.^[^ ^4^, ^20^, ^21^, ^22^, ^23^, ^24^, ^25^, ^26^, ^27^, ^28^, ^29^, ^30^ ^]^ Only recently the liquid crystalline SD^[^ ^31^, ^32^ ^]^ and SP^[^ ^33^ ^]^ structures (**Figure** 1e,f and **2**d) have been discovered as bottom‐up self‐assembled thermodynamic equilibrium structures of specifically designed polyphilic block‐molecules,^[^ ^34^, ^35^, ^36^, ^37^, ^38^ ^]^ These so‐called bolapolyphiles (BPs, see for example Figure 2a,b, combining three incompatible building blocks, a rigid rod‐like polyaromatic core, polar glycerol end groups and non‐polar flexible side chains) are known to self‐assemble into a huge number of new and complex LC phase morphologies,^[^ ^39^, ^40^, ^41^, ^42^, ^43^, ^44^, ^45^ ^]^ not achievable with conventional rod‐like or disc‐like LC molecules.^[^ ^46^, ^47^ ^]^ Among them are numerous honeycomb phases composed of prismatic cells with different polygonal shapes,^[^ ^48^, ^49^, ^50^, ^51^, ^52^, ^53^, ^54^, ^55^, ^56^, ^57^, ^58^, ^59^, ^60^ ^]^ lamellar phases,^[^ ^61^ ^]^ and coaxial rod‐bundle phases.^[^ ^62^, ^63^ ^]^ Some BPs form entirely new types of self‐assembled soft‐matter structures, for example, the segmented network phases with cubic or noncubic space groups. These are formed by bundles of parallel aligned polyaromatic rods, interconnected by spheres involving the nanosegregated glycerol end‐groups and the space between the networks is filled by the flexible aliphatic side chains (Figure 2c,d). Examples are the DG,^[^ ^63^, ^64^ ^]^ DD^[^ ^65^, ^66^ ^]^ and I‐WP networks (WP stands for “wrapped package” and “I” identifies the body centered cubic lattice),^[^ ^67^ ^]^ the A15 like tetrahedral network with *Pm* $\bar{3}$ *n* space group,^[^ ^68^, ^69^ ^]^ as well as the abovementioned SD and SP phases.^[^ ^33^, ^34^ ^]^

For these networks the side‐chain volume (*V*) and its development depending on the distance from the coaxial rod‐bundles (*r*) has a significant influence on the type of network structure being formed, as shown by the (*dV*/*dr*) = *f*(*r*) curves in Figure 1g.^[^ ^33^ ^]^ Overall, there is a sequence P → D → G with growing chain volume along the side chains. Second, the single‐network phases require much longer side chains with larger chain diameter than the corresponding double network structures (Figure 1g). This concept allowed the design of the SD^[^ ^31^, ^32^ ^]^ and SP phases.^[^ ^33^ ^]^ However, the SG structure, predicted for very large side chains, has not been achieved by the bottom‐up strategy yet, though it is of special interest as it exhibits unique properties, including complete photonic bandgap,^[^ ^70^ ^]^ intrinsic chirality,^[^ ^71^ ^]^ circular dichroism, and negative light diffraction, making it especially useful for optical applications, as well as it is applied by nature in numerous biophotonic structures.^[^ ^3^, ^4^, ^72^, ^73^, ^74^ ^]^

**Scheme 1:**
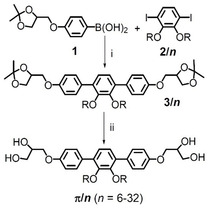
Synthesis of compounds ***π*** **/** ***n***. Reagents and conditions: a) [Pd(PPh_3_)_4_], sat. NaHCO_3_‐sol., THF, reflux, and b) HCl (10%), THF, MeOH, reflux.

Herein we report the soft self‐assembly of a series of *π*‐shaped *p*‐terphenyl‐based BPs ***π*** **/** ***n***, with two side‐by‐side arranged side chains (**Scheme** **1**), covering a superwide chain‐length range from *n* = 6–32 (**Table** **1**), and compare them with the self‐assembly of the related T‐shaped and K‐shaped molecules shown in Figure 2a ^[^ ^39^, ^40^, ^41^, ^75^ ^]^ and Figure 2b.^[^ ^31^ ^]^ A series of seven complex LC phases including DG and SD was found depending on side‐chain length, side‐chain structure, and temperature. However, instead of the expected transition to SG, the complete loss of LC phases was observed upon further chain elongation beyond *n* = 30. It is hypothesized that the SD represents the single network with the smallest possible valence of the nodes (*ν* = 4), while the SG with only *ν *= 3 would require the stabilization by interpenetration with a second network.

**Table 1:** Transition temperatures (*T*/°C) and associated enthalpy values ([Δ*H*]/kJ mol^−1^) of compounds ***π*** **/** ***n*** as measured on heating (*H*) and cooling (*C*).

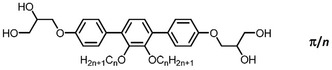					
Comp.a)	*n*	Phase transitions	*a,b*/[nm] (*T*/°C)	*n*_cell_	*n*_wall_/*n*_bundle_
***π*** **/6**	6	H: Cr 131 [6.3] Col_rec_/*p*2*mm* 139 [0.5] Col_squ_/*p*4*mm* [5.2] 155 Iso	2.55 (145)	3.2	1.59
		C: Iso 151 [‐5.4] Col_squ_/*p*4*mm* 136 [‐0.7] Col_rec_/*p*2*mm* 125 [‐6.1] Cr	2.33, 2.90 (135)	3.3	1.66
***π*** **/8**	8	H: Cr 130 [9.6] Col_squ_/*p*4*mm* 162 [8.9] Iso	2.57 (140)	2.9	1.44
		C: Iso 160 [‐8.8] Col_squ_/*p*4*mm* 124 [‐9.4] Cr			
***π*** **/9**	9	H: Cr 125 [9.6] Col_squ_/*p*4*mm* 149 [6.3] Iso	2.58 (145)	2.7	1.35
		C: Iso 146 [‐5.7] Col_squ_/*p*4*mm* 120 [‐9.1] Cr			
***π*** **/10**	10	H: Cr_1_ 69 [26.9] Cr_2_ 114 [7.6] Col_rec_^Z^/*c*2*mm* 139 [5.8] Iso	15.3, 6.16 (130)	36.4	n.d.c)
		C: Iso 137 [‐5.6] Col_rec_^Z^/*c*2*mm* 98 [‐6.6] Cr			
***π*** **/12**	12	H: Cr 88 [6.2] Col_rec_^Z^/*c*2*mm* 134 [5.6] Iso	15.72, 6.28 (119)	34.8	1.7/9.3d)
		C: Iso 131 [‐5.6] Col_rec_^Z^/*c*2*mm* 59 [‐4.2] Cr			
***π*** **/14**	14	H: Cr 85 [2.6] Col_hex_/*p*6*mm* 118 [5.7] Iso	4.15 (100)	4.9	1.7
		C: Iso 113 [‐5.3] Col_hex_/*p*6*mm* 61 [‐4.3] Cr			
***π*** **/16**	16	H: Cr 94 [6.3] Col_hex/_ *p*6*mm* 109 [3.2] Cub_net_/*Ia* $\bar{3}$ *d* 135 [1.6] Iso	7.64 (105)	297	12.4
		C: Iso 114 [‐1.0] Cub_net_/*Ia* $\bar{3}$ *d* 94 [‐3.5] Col_hex_/*p*6*mm* 85 [‐6.1] Cr	4.20 (95)	4.7	1.6
***π*** **18** ^[^ ^64^ ^]^	18	H: Cr 93 [70]^*b*^ Lam_Sm_/*p*2*mm* 97 [9.5] Cub_net_/*Ia* $\bar{3}$ *d* 128 [1.1] Iso	7.76 (100)	290	12.1
		C: Iso 120 [‐0.5] Cub_net_/*Ia* $\bar{3}$ *d* 84 [‐9.7] Lam_Sm_/*p*2*mm* 61 [‐70.1] Cr	2.36, 3.27 (75)	2.2	2.2
***π*** **/20**	20	H: Cr 95 [64.5] Cub_net_/*Fd* $\bar{3}$ *m* 129 [1.5] Iso	6.30 (100)	154	9.5
		C: Iso 123 [‐1.3] Cub_net_/*Fd* $\bar{3}$ *m* 69 [‐86.0] Cr			
***π*** **/22**	22	H: Cr 99 [99.7] Cub_net_/*Fd* $\bar{3}$ *m* 150 [2.9] Iso	6.29 (120)	136	8.5
		C: Iso 147 [‐2.8] Cub_net_/*Fd* $\bar{3}$ *m* 74 [‐106.5] Cr			
***π*** **/24**	24	H: Cr 99 [100.5] Cub_net_/*Fd* $\bar{3}$ *m* 151 [3.4] Iso	6.21 (120)	123	7.7
		C: Iso 147 [‐3.1] Cub_net_/*Fd* $\bar{3}$ *m* 78 [‐109.5] Cr			
***π*** **/26**	26	H: Cr 99 [103.8] Cub_net_/*Fd* $\bar{3}$ *m* 149 [2.8] Iso	6.22 (120)	117	7.3
		C: Iso 145 [‐2.8] Cub_net_/*Fd* $\bar{3}$ *m* 78 [‐110.8] Cr			
***π*** **/30**	30	H: Cr 106 [119.0] Cub_net_/*Fd* $\bar{3}$ *m* [‐] 135^*c*^ Iso	6.22 (120)	106	6.6
		C: Iso 130 [‐]b) Cub_net_/*Fd* $\bar{3}$ *m* [‐133.4] 91 Cr			
***π*** **32**	32	H: Cr 108 [142.3] Iso	–	–	–
		C: Iso 94 [‐153.2] Cr			

a) Data of the second DSC heating and cooling curves (10 K min^−1^, peak temperatures), for compounds ***π*** **/18**–***π*** **/22** the first DSC heating cycles were used; abbreviations: Cr, Cr_1_, Cr_2_ = crystalline solids, Col_squ_/*p*4*mm* = square LC honeycomb, Col_rec_/*p*2*mm* = rectangular LC honeycomb, Col_rec_^Z^/*c*2*mm* = zeolite‐like columnar LC phase composed of pentagonal and filled octagonal cells (see Figure 4c), Col_hex_/*p*6*mm* = hexagonal LC honeycomb, Lam_Sm_/*p*2*mm* = correlated lamellar LC phase with non‐centered lattice, Cub_net_/*Ia* $\bar{3}$ *d* = segmented double gyroid cubic network phase, Cub_net_/*Fd* $\bar{3}$ *m* = segmented single diamond cubic network phase, Iso = isotropic liquid, for DSC traces see (Figure S1, Supporting Information), for XRD data, see (Table S2, S4, S5, S7 and S9, Supporting Information), and for detailed structural data, see (Table S3, S6, S8 and S10, Supporting Information).

b) No transition enthalpy, therefore determined by microscopy.

c) Not determined, as no ED map was available.

d) Evaluated based on the ED map in Figure 4b, see Table S6, Supporting Information.

## 2 Results

### 2.1 Synthesis

The synthesis of compounds ***π*** **/** ***n*** is straightforward and follows the procedure reported for ***π*** **/18**,^[^ ^64^ ^]^ starting with the alkylation of 2,6‐diiodocatechol^[^ ^76^ ^]^ with *n*‐alkyl bromides, leading to the diethers **2/** ***n***, followed by Suzuki coupling^[^ ^77^ ^]^ with two equivalents of the protected and glycerol‐functionalized benzene boronic acid **1** ^[^ ^78^ ^]^ and deprotection of the two glycerol groups of the obtained bisacetonides **3/** ***n*** (Scheme 1).^[^ ^64^, ^79^ ^]^

### 2.2 Liquid Crystalline Self‐Assembly

**Figure 3:**
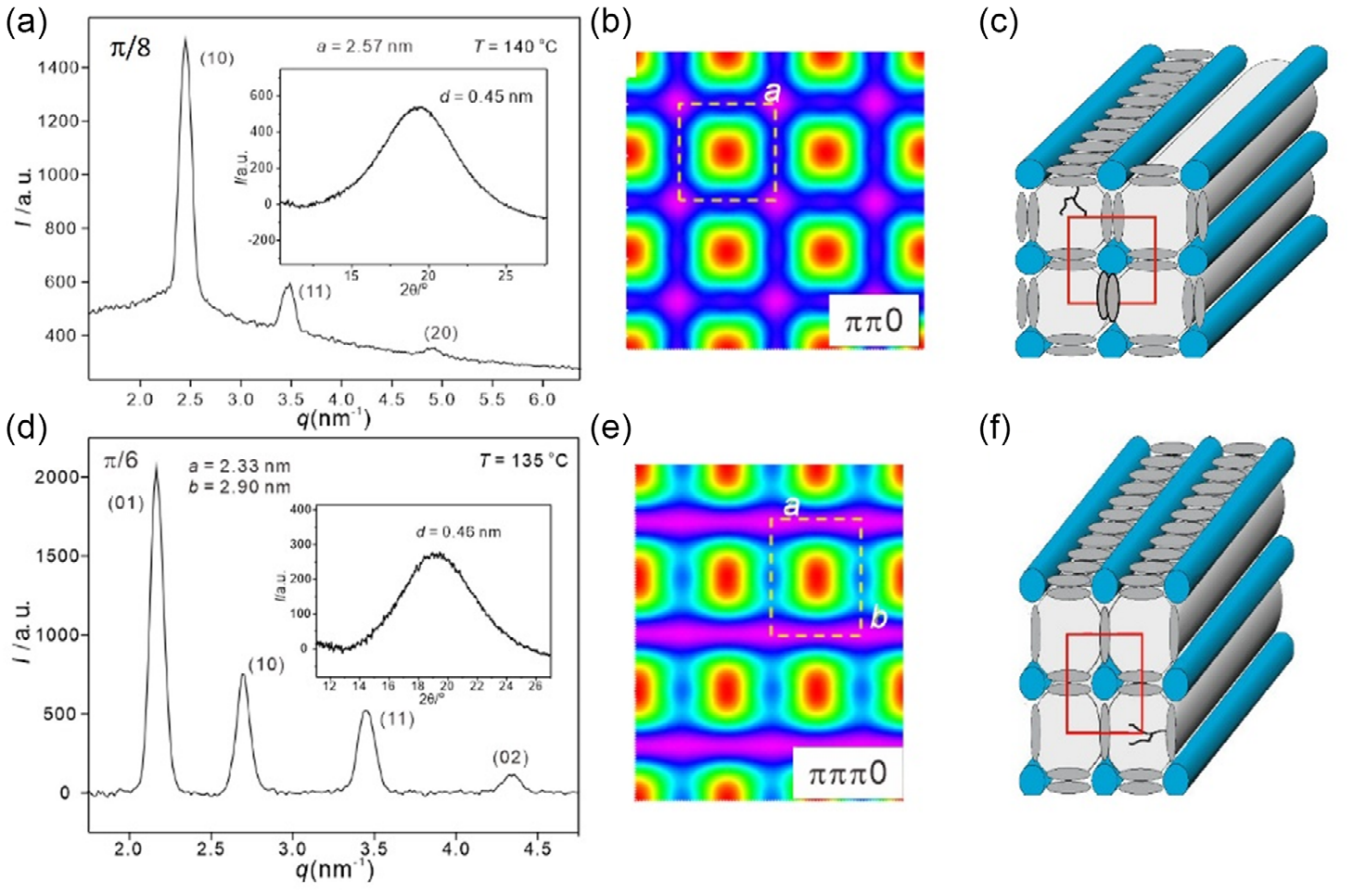
LC honeycomb phases of compounds ***π*** **/8** and ***π*** **/6.** a) SAXS and WAXS scans (inset), b) ED map (phase combination: *ππ*0, red = lowest ED, purple = highest ED), and c) model of the square honeycomb Col_squ_/*p*4*mm* phase of ***π*** **/8** at *T* = 140 °C, d) SAXS and WAXS scans (inset), e) reconstructed ED map (phase combination: *πππ*0), and f) model of the rectangular honeycomb Col_rec_/*p*2*mm* phase of ***π*** **/6** at *T* = 135 °C; for SAXS data of ***π*** **/9,** see Table S2 and Figure S3, Supporting Information, and for structural data of ***π*** **/6**–***π*** **/9,** see Table S3, Supporting Information.

All compounds ***π*** **/** ***n*** with *n* = 6–30 form at least one enantiotropic (thermodynamically stable and reversibly formed) birefringent or isotropic mesophase (Table 1). The LC nature of all mesophases is confirmed by the diffuse wide‐angle scattering (WAXS) with a maximum at *d* = 0.45–0.46 nm (see, e.g., insets in **Figure** **3**a,d) and in addition by the fluidity or viscoelasticity (cubic phases) of the samples, as observed by shearing under the polarizing optical microscopy (POM), both together indicating the rotational freedom and the nonfixed positions of the individual molecules. In the following subsections, the distinct types of LC self‐assembly of the compounds are described in the order of increasing side‐chain length, while the full sequence is discussed, analyzed, and compared with series of related compounds in Section 3.

### 2.3 Square and Rectangular Honeycombs (Col_squ_, Col_rec_)

On cooling between crossed polarizers (POM) from the isotropic liquid, compounds ***π*** **/6**, ***π*** **/8,** and ***π*** **/9** exhibit spherulitic optical textures with pseudoisotropic homeotropic areas as typical for uniaxial columnar LC phases (see Figure S2a, Supporting Information). Observations of the textures with a full‐wave retarder plate show that these mesophases have a negative birefringence, that is, that the slow optical axis, coinciding with the major intramolecular *π*‐conjugation pathway along the long axis of the *p*‐terphenyls is aligned perpendicular to the column long axis (see inset in Figure S2a,f, Supporting Information). Small‐angle X‐ray scattering (SAXS) experiments of the uniaxial mesophases of ***π*** **/6**–***π*** **/9** (see Figure 3a, S3, Supporting Information) show diffraction patterns as typical for a square lattice with *p*4*mm* plane group (*d*‐value ratio: 1: $\sqrt{2}$ :2) with the lattice parameter *a*_squ_ ≈ 2.55–2.58 nm (Col_squ_/*p*4*mm* phase). The lattice parameter does not change significantly by increasing the lateral chain volume, and this value is close to the maximum length of the molecules as measured between the two primary OH groups of the glycerol end groups (see Figure S7a, Supporting Information, *L*_mol_ = 2.6 nm) and is in line with a square honeycomb structure as additionally confirmed by the electron density (ED) map. The diffraction pattern and ED map of the *p*4*mm* phase of compound ***π*** **/8** are shown as examples in Figure 3a,b. The low‐ED regions (red), which are formed by the alkyl chains, have a square shape and are enclosed within a square net of high ED (purple), formed by the *p*‐terphenyls at the edges and the glycerol groups at the vertices. This indicates a square honeycomb formed by walls of the parallel aligned *p*‐terphenyls (Figure 3c) and held together at the edges by columns involving the glycerol end groups with highest cohesive energy density (CED) and the resulting square prismatic cells being filled by the flexible aliphatic side chains.

On further cooling compound ***π*** **/6**, having the shortest side chains among the synthesized compounds reveals an LC–LC transition at *T* = 136 °C on cooling (and at 139 °C upon heating), which is associated with a small enthalpy (Δ*H* ≈ 0.6 kJ mol^−1^). The former dark homeotropic areas of the Col_squ_/*p*4*mm* phase become birefringent with development of a typical stripe pattern (see Figure S2b, Supporting Information).^[^ ^32^, ^75^ ^]^ The SAXS pattern (see Figure 3d) changes abruptly at the phase transition and can be assigned to a noncentered rectangular lattice with *p*2*mm* plane group and the parameters *a*_rec_ = 2.3 and *b*_rec_ = 2.9 nm. The parameter *a*_rec_ is close to the shortest possible molecular length (*L*_mol_ = 2.1 nm), while *b*_rec_ is a bit longer than the maximal molecular length (*L*_mol_ = 2.6 nm, see Figure S7a, Supporting Information). This deformation of the square honeycomb to a rectangular (Figure 3e,f) has previously been observed in the series of compounds **T** **/** ***n*** and **K** **/** ***n*** for *n* = 8–12 and *n* = 6, respectively and has been discussed for these compounds in more detail as a result of partial chain stiffening and chain volume reduction at lower temperature^[^ ^32^, ^75^, ^80^ ^]^ (see Section S2.4, Supporting Information for more details).

### 2.4 Zeolite‐Like Pentagon–Octagon Tiling (Col_rec_^Z^)

**Figure 4:**
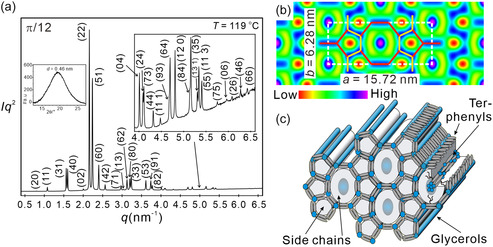
Zeolite‐like columnar phase (Col_rec_^Z^/*c*2*mm*) of compound ***π*** **/12.** a) SAXS pattern at *T* = 119 °C, as inset, WAXS scan at 120 °C, b) reconstructed ED map (phase combination 0ππ0π000) with the organization of the terphenyls shown as thin white and red lines, and c) structural model of the phase.

For the next compounds ***π*** **/10** and ***π*** **/12** with longer side chains an optically biaxial columnar phase is found by POM investigations (Figure S2c, Supporting Information). The SAXS pattern of ***π*** **/12**, as example, shows numerous Bragg reflections, which can be indexed to a centered rectangular lattice with space group *c*2*mm* (reflection conditions *h*0: *h* = 2*n*, 0*k*: *k* = 2*n*, *hk*: *h* + *k* = 2*n*, see **Figure** **4**a, and large lattice parameters of *a*_rec_ ≈ 15.3 and *b*_rec_ ≈ 6.15 nm), by far exceeding the molecular dimensions and indicating a complex superlattice structure. Optical investigations confirm negative birefringence (inset in Figure S2c, Supporting Information), in line with the honeycomb phases. As obvious from the reconstructed ED map in Figure 4b and from previous investigations with compounds **K** **/** ***n*** ^[^ ^32^, ^60^ ^]^ the structure consists of alternating ribbons of pentagons and octagons where adjacent pentagons show an antiparallel orientation to each other (see Figure 4c). Each pentagonal channel is filled with alkyl chains and additional *p*‐terphenyl walls separate these pentagonal ribbons which leads to octagonal cells. The space within the octagonal channels is filled by alkyl chains too, but to fill the space in these octagonal cells completely, additional columns of parallel aligned rod‐bundles fill the centers of these cells coaxial with the *c*‐axis (“zeolite‐like” Col_rec_^Z^/*c*2*mm* phase). As described in previous work,^[^ ^32^, ^60^ ^]^ the reason for the formation of this honeycomb with complex tiling pattern is a steric frustration. While the side‐chain volume would require hexagonal cells (for **T** **/** ***m*** Col_hex_ is found for *m* = 16–22,^[^ ^75^ ^]^ for compounds ***π*** **/** ***n*** with a similar chain volume, but shorter chains for *n* = 10, 12), the distance between the *p*‐terphenyl core and the ends of the side chains requires a significant chain stretching in order to fill the centers of the hexagonal cells, which is entropically unfavorable. Therefore, the smaller pentagons are formed and the steric frustration caused by the excess chain volume leads to the formation of additional larger cells and development of a complex superstructure due to the emerging geometric frustration in this packing mode.

### 2.5 Hexagonal Honeycomb (Col_hex_)

**Figure 5:**
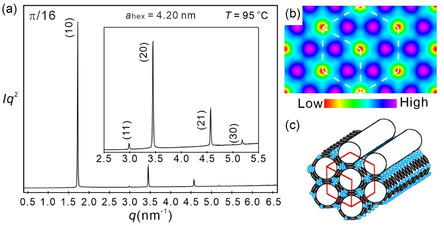
Hexagonal honeycomb LC phases of compounds ***π*** **/16**: a) SAXS scan at *T* = 95 °C, b) ED map (phase combination: *πππππ*), and c) structural model.

In the whole LC temperature range of compound ***π*** **/14** a uniaxial LC phase with spherulitic texture and negative birefringence is observed (see Figure S2d, Supporting Information). The same phase is found for ***π*** **/16** as a low‐temperature phase below an optical isotropic mesophase. For compound ***π*** **/16** the SAXS pattern is characterized by four sharp small‐angle reflections (**Figure** **5**a) with a *d*‐value ratio of 1: $\sqrt{3}$ :2: $\sqrt{7}$ which can be indexed as a columnar phase with *p*6*mm* plane group and a lattice parameter of *a*_hex_ ≈ 4.2 nm (Col_hex_/*p*6*mm*). The ED map in Figure 5b shows a hexagonal honeycomb with a side length of *a*_hex_/√3 = 2.4 nm fitting well with the length of the bolaamphiphilic core. The number of molecules per unit cell with a height of 0.45 nm is *n*_cell_ = 4.9 for ***π*** **/14** and 4.7 for ***π*** **/16**, corresponding to hexagonal honeycombs with *n*_wall_ = 1.6 (***π*** **/14**) and 1.5 (***π*** **/16**) molecules in the cross section of the individual honeycomb walls (Figure 5c).

### 2.6 Double Gyroid (DG) Network Phase

**Figure 6:**
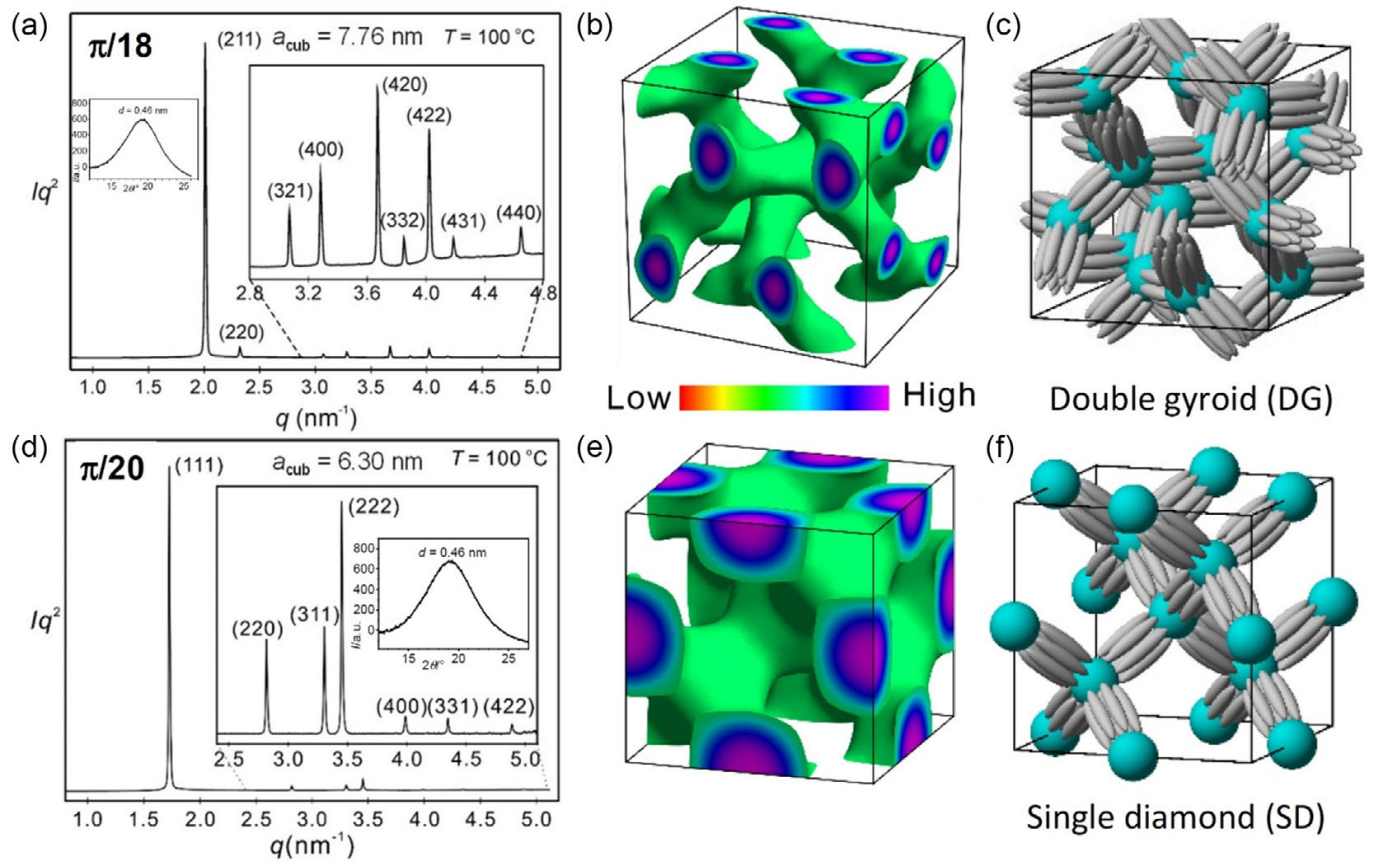
a–c) Cub_net_/*Ia* $\bar{3}$ *d* phase of compound ***π*** **/18** and^[^ ^64^ ^]^ d–f) Cub_net_/*Fd* $\bar{3}$ *m* phase of ***π*** **/20.** (a) *q* scan of ***π*** **/18** at *T* = 100 °C with inset showing the WAXS at 120 °C, (b) reconstructed ED map (phase combination: *ππ*0*ππ*0000), only areas of high ED (aromatics + glycerol groups) are shown, representing one unit cell, (c) model of molecular organizations, (d) *q* scan of ***π*** **/20** at *T* = 100 °C and inset with WAXS at 110 °C, (e) reconstructed ED map (phase combination: 0*π*0000*π*), and (f) model of molecular organizations. A 2 × 2 × 2 ED map with histogram is shown in Figure S5, Supporting Information, and the ED reconstruction process is described in Figure S6, Supporting Information.

For ***π*** **/14** the Col_hex_/*p*6*mm* phase represents the only mode of LC self‐assembly, while for the next even numbered homologue ***π*** **/16** a transition to an optically isotropic and viscous mesophase takes place on heating, before the transition to the highly fluid isotropic liquid (Iso) is observed. This transition is indicated in the differential scanning calorimetry (DSC) heating traces by an endotherm with Δ*H* = 1.6 kJ mol^−1^, showing a delay of 15 K upon cooling with a relatively high rate of 10 K min^−1^ (see Table 1 and Figure S1, Supporting Information). This temperature difference is larger if compared to the honeycomb–honeycomb transitions, showing no or only a very small hysteresis. Nevertheless, kinetic effects during the transition between 2D and 3D lattices are still comparatively small and the structure formation is for these low‐molecular weight LCs determined by thermodynamics. This is in stark contrast to block copolymers where kinetic effects play a much larger role due to their much higher viscosity. The SAXS scan can be assigned to the *Ia* $\bar{3}$ *d* cubic space group (ratio of *d*‐values: $\sqrt{6}:\sqrt{8}:\sqrt{14}:\sqrt{16}:\ldots$) with *a*_cub_ = 7.64 nm (see Table S7, Supporting Information). The same Cub_net_/*Ia* $\bar{3}$ *d* phase is observed for compound ***π*** **/18** in the temperature range between 97 °C and the transition to the isotropic liquid state at 128 °C (**Figure** **6**a). The ED map of the Cub_net_/*Ia* $\bar{3}$ *d* phase of ***π*** **/18** (see Figure 6b) shows two interpenetrating networks with DG structure.^[^ ^64^ ^]^ Each network is formed by 12 struts which are connected by three‐way junctions. The ED map in Figure 6b shows only the high‐ED networks (green surface) involving the glycerol groups and the *p*‐terphenyls, while the low‐ED continuum formed by the alkyl side chains is not shown for clarity. Within the networks the polar and high‐CED glycerol groups are located at the junctions and the *p*‐terphenyls are organized in bundles (the struts), which consist of ≈12 parallel aligned molecules (*n*_bundle_ = *n*_cell_/24, see Table S8, Supporting Information). This means that ≈36 glycerols are organized in each of the glycerol domains at the three‐way junctions. The distance between neighboring junction points (in the middle of the glycerol domains) was calculated to *a*_cub_/(2·$\sqrt{2}$) ≈ 2.7 nm.^[^ ^81^ ^]^ This value is close to the molecular length in the most stretched conformation, meaning that exactly one bundle of molecule forms the struts interconnecting the polar domains at the junctions (Figure 6c).^[^ ^64^ ^]^ That this distance slightly exceeds the maximum molecular length of *L*_mol_ = 2.6 nm is due to the significant size of the glycerol domains.

### 2.7 Correlated Layer Phase (Lam_Sm_)

**Figure 7:**
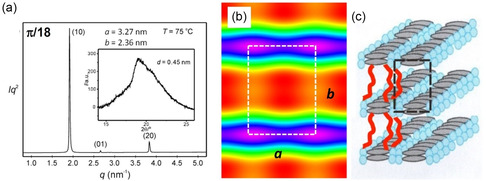
Correlated layer phase of compound ***π*** **/18.** a) *q* scan at *T* = 75 °C with inset showing the WAXS, b) reconstructed ED map (phase combination: *ππ*0, red = lowest ED, purple = highest ED), and c) model of organization of the molecules.

For compound ***π*** **/18** a transition to a birefringent mesophase with spherulitic texture is observed by POM upon cooling from the cubic phase at *T* = 84 °C (see Figure S2e, Supporting Information), which is also evident from the temperature dependence of the SAXS pattern (Figure S4, Supporting Information). The *q* scan of the SAXS region at 75 °C (**Figure** **7**a) shows three sharp scatterings that can be indexed to a noncentered *p*2*mm* lattice (*a*_rec_ = 2.4, *b*_rec_ = 3.3 nm). The reconstructed ED map (see Figure 7b) shows ribbons of high (blue/purple) and low ED (red) and indicates the formation of a lamellar phase with AA stacking of adjacent layers (Lam_Sm_/*p*2*mm*). In the lamellar phase the rod‐like cores arrange perpendicular to the layer normal and assume orientational order within the layers. The segregation of the glycerol groups, which form strings of hydrogen bonding network, leads to an additional long‐range positional order in the layers (Lam_Sm_ phase). The long range  correlation of the interlayer and intralayer periodicities gives rise to a 2D lattice parallel to layer normal and in‐plane nematic director (Figure 7c). Each unit cell defined by *a*_rec_, *b*_rec_, and a height of *h* = 0.45 nm along the *c*‐direction contains two molecules which are “back‐to‐back” organized in the layers (see Table 1 and S7, Supporting Information). The parameter *a*_rec_ = 2.4 nm refers to the molecular length and *b*_rec_ = 3.3 nm to the interlayer distance. The remaining gap of *b*_rec_ − 0.9 = 2.4 nm (0.9 nm is the width of a layer formed by two back‐to‐back arranged *p*‐terphenyls) is filled with the alkyl side chains (low ED, red). The length of the alkyl chains with *n* = 18 is about 2.5 nm; hence, the side chains are assumed to be aligned fully interdigitated and preferentially parallel or only slightly tilted to the layer normal.

### 2.8 SD Network Phase

Compounds ***π*** **/20**–***π*** **/30** show exclusively an optically isotropic cubic mesophase, that is, the lamellar phase is removed. However, the SAXS pattern (Figure 6d) differs significantly from the DG phase (see Figure 6a). In the SAXS pattern the *d*‐value ratio is $\sqrt{3}:\sqrt{8}:\sqrt{11}:\sqrt{12}:\sqrt{16}:\sqrt{19}:\sqrt{24}$ (see Table S8, Supporting Information), which can be assigned to the cubic space group *Fd* $\bar{3}$ *m* with *a*_cub_ = 6.2–6.3 nm for all 5 compounds (Cub_net_/*Fd* $\bar{3}$ *m*). The ED map, calculated from all seven SAXS peaks of ***π*** **/20,** is shown in Figure 6e and confirms the SD phase formed by a single ED‐rich network (purple, blue with green surface) that is embedded in a low‐ED alkyl chain continuum (not shown for clarity). As obvious from this ED map, each unit cell contains 16 struts interconnected by the clearly visible tetrahedral four‐way junctions provided by domains of the glycerol groups (see also Figure S5, Supporting Information). Thus nine molecules are organized side by side in the lateral cross section of the bundles of parallel aligned rods (Table 1). The distance between two adjacent junctions can be calculated by *d*_nodes_ = ($4/\sqrt{3}$)/*a*_cub_ = 2.7 nm^[^ ^81^ ^]^ which is the same as in the Cub_net_/*Ia* $\bar{3}$ *d* (DG) phase and meaning that the distance between the junctions corresponds to a single‐molecular length (Figure 6f). The distance *d*_nodes_ slightly exceeds *L*_mol_ due to the significant size of the polar spheres involving 36 glycerol groups, which is close to this number in the Cub_net_/*Ia* $\bar{3}$ *d* (DG) phase. As the lattice parameter remains almost constant, with increasing side‐chain volume, the number of molecules organized in the unit cell (*n*_cell_) as well as in the rod bundles decreases to *n*_bundle_ = 6.6, and the number of glycerols at the junctions decreases to 26 for ***π*** **/30** (see Table S9, Supporting Information).

**Figure 8:**
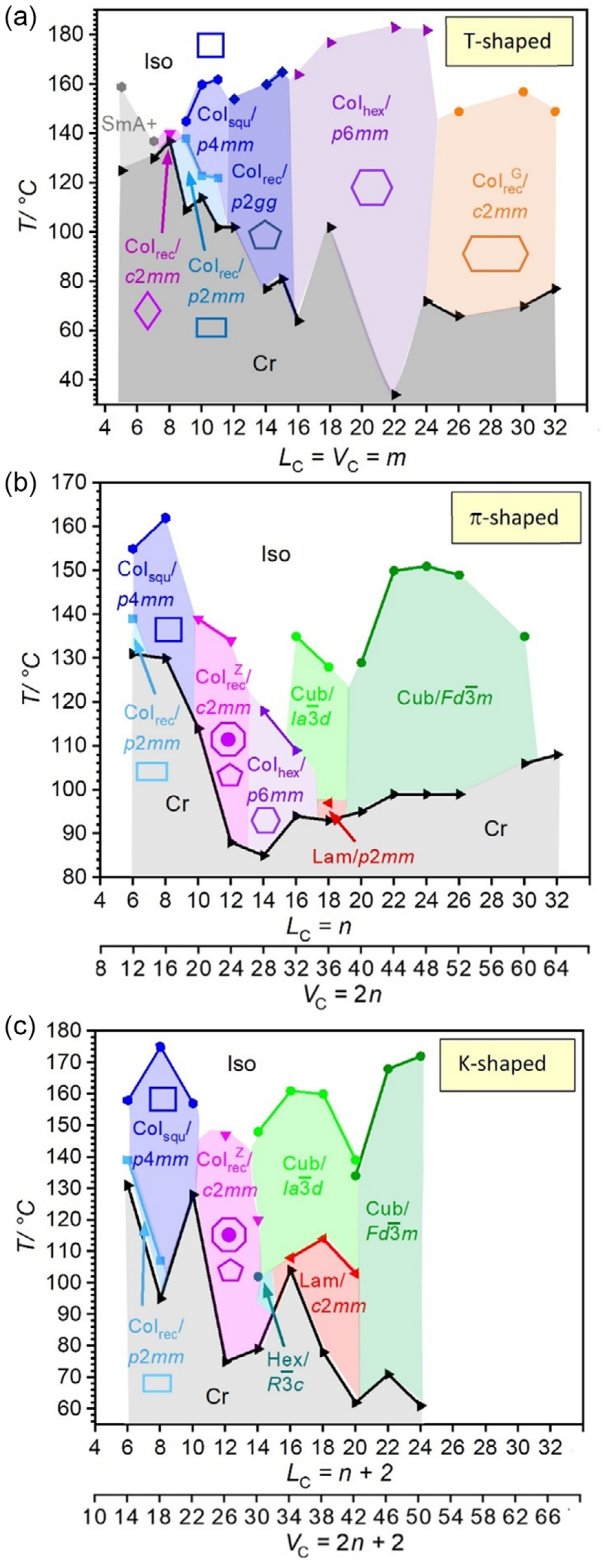
Schematic graphics of the development of the distinct LC phases and their transitions depending on the distance between *p*‐terphenyl core and end of the side chain, given as the number of carbon atoms *L*_C_, the total number of carbon atoms in the side chains *V*_C_, and temperature for the series. a) **T** **/** ***m***,^[^ ^75^, ^82^ ^]^ b) compounds ***π*** **/** ***n,*** and c) **K** **/** ***n*** (note that *R* $\bar{3}$ *c* is monotropic with respect to Col_rec_^Z^/*c*2*mm*)^[^ ^32^ ^]^ (recorded on heating; see Table 1 and refs. [32, 75] for numerical values); for abbreviations, see Table 1; the SmA+ phase^[^ ^49^ ^]^ has the terphenyls organized parallel to the layer normal and is therefore not considered here.

Interestingly, the cubic phase is completely lost for ***π*** **/32**, the compound with the longest side chains, which directly melts with formation of a low‐viscous isotropic liquid. Even upon cooling, no indication of a monotropic mesophase was observed before crystallization takes place at 94 °C (Figure S1, Supporting Information). Overall, in the series ***π*** **/20–** ***π*** **/32,** the Cub_net_/*Fd* $\bar{3}$ *m* (SD)‐Iso transition temperature first increases from ***π*** **/20** to ***π*** **/22**, reaches a plateau, and remains almost constant for ***π*** **/24–** ***π*** **/28** before it drops again for ***π*** **/30** and has completely vanished for ***π*** **/32** (Table 1 and **Figure** **8**b). There is a similar trend for the SD‐Iso transition enthalpies, which increases from 1.5 kJ mol^−1^ for ***π*** **/20** to 3.4 kJ mol^−1^ for ***π*** **/24** and then drops upon further chain elongation until it becomes invisible (<0.01 kJ mol^−1^) for ***π*** **/30** just before the SD phase completely disappears for ***π*** **/32**, for which the capability of LC self‐assembly is lost. With other words, the expected transition SD → SG upon side‐chain elongation (Figure 1g) does not take place.

## 3 Discussion

### 3.1 Effects of Side‐Chain Structure on IMDS

In Figure 8 the phase sequence of the series of *π*‐shaped compounds ***π*** **/** ***n*** is compared with the related K‐ and T‐shaped compounds **K** **/** ***n*** ^[^ ^32^, ^60^ ^]^ and **T** **/** ***m***.^[^ ^75^, ^82^ ^]^ There is a general trend for the mesophase stability (LC‐to‐Iso transition temperatures) to decrease in the order **T** **/** ***m*** > **K** **/** ***n*** > ***π*** **/** ***n***, meaning that two instead of only one side chain distort the packing of the molecules a bit more than chain branching. However, these effects are small and all three series can form LC phases over wide temperature and chain length ranges. In the diagrams *L*_C_ gives the number of *C*‐atoms between the *p*‐terphenyl core and the most distant end of the side chain(s), which equals *L*_C_ = *m* for compounds **T** **/** ***m***, *L*_C_ = *n* for ***π*** **/** ***n,*** and it is *L*_C_ = *n *+ 2 for **K** **/** ***n***. The side‐chain volume, *V*_C_, shown on the lower *x*‐axes, is the total number of CH_2_ equivalent. There is a huge effect of *L*_C_ and *V*_C_ on the phase stability and the structure of the soft self‐assembled LCs which differ in the crystallographic space groups and the curvature of the involved intermaterials dividing surfaces (IMDSs). While for binary amphiphiles there is a single uniform type of IMDS between only two different types of domains, leading to the well‐known sequence lamellae → networks → columns → spheres with increasing curvature,^[^ ^6^, ^7^, ^8^ ^]^ for polyphiles the situation is more complicated, as several interfaces between the distinct domains play a role: rods/alkyls, glycerols/alkyls, rods/glycerols, alkyls/glycerols. For the compounds under consideration these four interfaces can be combined to only two, representing the most important for the following discussion. The IMDS^R^ is the interface between the alkyl chain domains (R) and the combined domains involving the polar glycerols and the rigid *p*‐terphenyl rods forming the aggregates (honeycombs or networks and columns), while the IMDS^P^ is the curvature between the polar glycerol domains (P) forming either columns or spheres embedded between the hydrocarbon segments, that is, the alkyl chains and the *p*‐terphenyls. In contrast to flexible amphiphiles/polyphiles and their macromolecular analogues (block copolymers) where the IMDS curvature and the Flory Huggins parameter represent the key factors determining the mode of self‐assembly, the situation is more complicated for the BPs considered here. The rigidity and linear shape of one of the building blocks and its well‐defined length have a stark effect on the self‐assembly by providing orientational order and limiting the available degree of curvature of the IMDS^R^ around these units to discrete values. In this respect there are some relations to rod‐coil polymers.^[^ ^83^, ^84^ ^]^

### 3.2 Honeycomb–Gyroid Transition

For the previously investigated series **T** **/** ***m*** the sequence of phases involves exclusively honeycombs, ranging from rhombic, rectangular, and square via pentagonal and regular hexagonal to giant hexagonal cells (Col_rec_^G^/*c*2*mm*), the latter having two walls of the hexagons formed by end‐to‐end pairs of *p*‐terphenyl rods (Figure 8a).^[^ ^53^, ^82^ ^]^ The SmA+ phase with only short range ordered alkyl chain domains within the layers is a precursor of the honeycombs which is described earlier and not considered here.^[^ ^49^, ^78^ ^]^ In this series the IMDS^R^ curvature remains positive, that is, the aliphatic domains are surrounded by the honeycomb walls formed by the terphenyls and glycerols providing the higher CED, and the IMDS^R^ curvature decreases with the number of sides of the polygonal cells, but it never reaches zero for the investigated chain lengths. In all these honeycombs, the high‐CED glycerols are organized in columns; hence, the IMDS^P^ curvature is negative and does not change considerably in the whole series of compounds **T** **/** ***m.***

For the compounds ***π*** **/** ***n*** two linear alkyl chains with identical length are organized side by side, thus doubling the chain volume *V*_C_ at a given length *L*_C_. Hence, the available chain volume exceeds that achievable with the *T*‐shaped molecules **T** **/** ***m*** with the same length and, in addition, there is a doubled effective side‐chain cross‐sectional area for the series **K** **/** ***n*** and ***π*** **/** ***n***. Therefore, there is a stronger tendency to decrease the positive IMDS^R^ curvature and the honeycombs can be replaced by a lamellar phase (Lam_Sm_/*p*2*mm*) with zero IMDS^R^ curvature and two different network phases (Cub_net_/*Ia* $\bar{3}$ *d* and Cub_net_/*Fd* $\bar{3}$ *m*) with negative IMDS^R^ curvature for the compounds with longest chains (Figure 8b). This difference in side‐chain volume and side‐chain diameter also leads to major differences between the series **T** **/** ***m*** and ***π*** **/** ***n***. Most remarkable is the formation of the zeolite‐like Col_rec_^Z^/*c*2*mm* phase (***π*** **/12–** ***π*** **/13**, see Figure 4), obviously replacing the monohedral pentagonal and hexagonal honeycomb phases of compounds **T** **/12–** **T** **/24**. This self‐assembled LC structure is unique, because it combines the positive IMDS^R^ curvature of the honeycomb framework with a negative provided by the rod bundles filling the larger octagonal cells (Figure 4c). If alternatively, the IMDS^P^ curvature of the glycerol domains is considered, the zeolite‐like phase can be regarded as a combined organization of polar glycerol columns (at the edges of the honeycomb cells) and polar spheres (the junctions along the columns of the rod bundles in the octagonal cells), being combined in a single uniform periodic structure. However, in contrast to the column + sphere^[^ ^85^, ^86^ ^]^ or network + sphere combinations,^[^ ^87^, ^88^ ^]^ being simulated^[^ ^87^ ^]^ or experimentally found^[^ ^88^ ^]^ in triblock copolymer morphologies, where columns/networks and spheres were formed by different materials, in the present case the columns and spheres, though having different degree of curvature, involve the same material (polar glycerols).

The suppression of any monohedral pentagon tiling in the series ***π*** **/** ***n*** is mainly due to the large side‐chain volume (requiring hexagonal and larger cells) in combination with a restricted side‐chain length, only being capable to reach the centers of the smaller square and pentagonal cells without the entropically unfavorable excessive chain stretching. Only for compounds ***π*** **/14** and ***π*** **/16** the side chains become sufficiently long that these chains can reach the centers of hexagonal cells and then hexagonal honeycombs can become sufficiently stable. At higher temperature, however, as the flexibility of the linear chains increases, the effective chain length is reduced, and the centers of the hexagons cannot be reached any more. Therefore, the hexagonal honeycomb is destabilized and lost for ***π*** **/14** at higher temperature. For the next homologue ***π*** **/16** the thermal chains expansion of the even longer chains removes the hexagonal honeycomb too, but in this case, the achieved chain volume is sufficient for development of a negative IMDS^R^ curvature leading to a transition to the DG network phase. The next compound ***π*** **/18** allows further increased curvature also at lower temperature and the hexagonal honeycomb is completely removed and replaced by the DG (Figure 8b).

**Figure 9:**
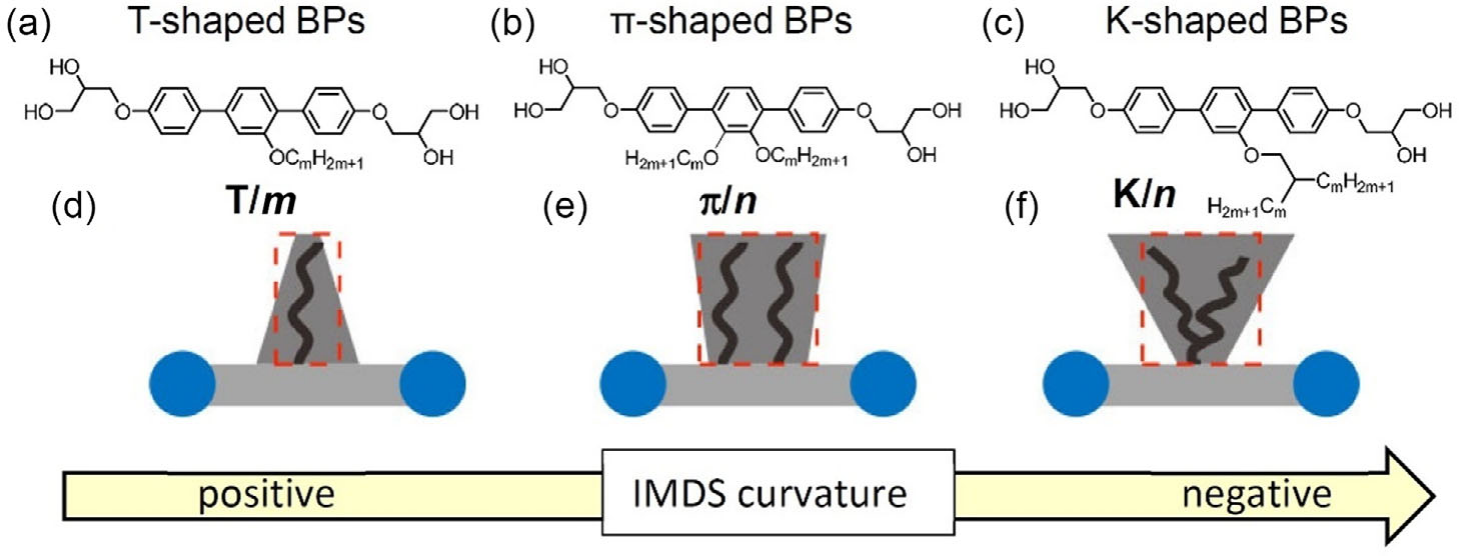
a–c) show the molecular structures of the different types of BPs under discussion. d–f) show the effective shapes assumed by the side chains during LC self‐assembly of the distinct types of BPs. Red boxes in (d–f) suggest the average volume of alkyl chains, for which ***π*** **/** ***n*** and **K** **/** ***n*** are almost same. The grey shadow represents the alkyl chain shape corresponding to the IMDS curvature.

Remarkably, the hexagonal honeycomb is not found in the series **K** **/** ***n*** (Figure 8c) because for the branched chains more conformational disorder is present and a larger entropic penalty for chain stretching than for the linear chains of the ***π*** **/** ***n*** compounds arises, destabilizing the hexagonal honeycomb even more (**Figure** **9**). The more disordered branched chains of compounds **K/** ***n*** thus lead to a direct transition from Col_rec_^Z^/*c*2*mm* to Cub_net_/*Ia* $\bar{3}$ *d*, removing the hexagonal honeycomb completely. The sequence Col_squ_/*p*4*mm*–Col_rec_^Z^/*c*2*mm*–Cub_net_/*Ia* $\bar{3}$ *d* upon chain elongation in the series **K** **/** ***n*** not only provides a smooth transition from positive to negative IMDS^R^ curvature, but it is also associated with a smoothly increasing degree of negative IMDS^P^ curvature from polar columns (Col_squ_/*p*4*mm*) via a mixed structure combining polar columns with spheres (Col_rec_^Z^/*c*2*mm*) to one with exclusively spheres at the junctions of the DG (and the following SD network). Thus, the sequence monohedral honeycomb, Col_rec_^Z^/*c*2*mm*–DG–SD, can be considered as the generic phase sequence of sticky rods with bulky lateral tethers.^[^ ^89^ ^]^ Under this aspect the hexagonal honeycomb occurring between Col_rec_^Z^/*c*2*mm* and DG in the series ***π*** **/** ***n*** can be considered as a discontinuity in this smooth development of the IMDS^P^ curvature by re‐entrance of a monohedral honeycomb with all glycerols in columns, thus temporarily removing all spheres before they emerge again in the DG. This is attributed to the linear side chains providing some similarity to the T‐shaped compounds. As shown in Figure 9 the tendency toward formation of negative IMDS curvature increases from **T** **/** ***m*** to ***π*** **/** ***n*** due to lateral chain expansion (single versus double chains) and to **K** **/** ***n*** (e.g., removal of Col_hex_) by additional lateral expansion due to increased conformational disorder.

### 3.3 Lamellar Phases

In both series ***π*** **/** ***n*** and **K** **/** ***n,*** a lamellar phase (Lam_Sm_, terphenyls parallel to the layers) is observed on cooling below the DG, in line with reduced side‐chain mobility reducing the IMDS^R^ curvature to zero around the chain length/volume driven transition from positive to negative curvature (Figure 8b,c). The lamellar range is larger for the series **K** **/** ***n*** (**K** **/13–** **K** **/18**)^[^ ^32^ ^]^ while in the series ***π*** **/** ***n*** only ***π*** **/18** forms a lamellar phase. The expansion of the Lam range toward lower side‐chain volume is again assumed to be due to the increased chain disorder of the branched chains in compounds **K** **/** ***n*** (Figure 9), leading to a stronger reduction of the positive IMDS^R^ curvature. In both series the Lam_Sm_ phase can only be found below the DG and not below SD. Thus, the generic phase sequence at low temperature becomes monohedral honeycomb, Col_rec_^Z^/*c*2*mm*–Lam_Sm_–SD upon side‐chain expansion with the option of formation of additional small ranges of a hexagonal honeycomb or a (monotropic) distorted DG (*R* $\bar{3}$ *c*)^[^ ^32^ ^]^ between Col_rec_^Z^/*c*2*mm* and Lam_Sm_. However, the Lam_Sm_ phase of ***π*** **/18** is different from that found for compounds ***K*** **/13–** ***K*** **/18**,^[^ ^32^ ^]^ as it has a simple (*p*2*mm*, no shift between the layers along *b*) instead of a centered 2D lattice (*c*2*mm*, shifted by *b*/2) due to an AA instead of AB correlation between the layers.^[^ ^61^ ^]^ This difference might be also attributed to the different chain conformation in the series ***π*** **/** ***n*** and **K** **/** ***n***. The parallel packing of the linear chains of compounds ***π*** **/** ***n***, being predominately aligned parallel to the layer normal, might improve the side‐by‐side coupling between the *p*‐terphenyls in adjacent layers, while the alkyl chains in the space between adjacent glycerol columns remain less ordered (chain folding is required to fill this space) as indicated by the slightly reduced ED in these regions (orange instead of red, see Figure 7b). The presence of more and less ordered chains is supported by the narrower and nonsymmetric shape of the WAXS scattering in the Lam_Sm_/*p*2*mm* phase of ***π*** **/18** (inset in Figure 7a). The segregation between more and less ordered domains along direction *a* supports the AA coupling of the lamellae.^[^ ^90^ ^]^ In contrast, the enhanced flexibility of the branched chain of compounds **K** **/** ***n*** (almost symmetric diffuse WAXS)^[^ ^32^ ^]^ removes this driving force for layer correlation and allows an easier space filling in the voids around the glycerols by the alkyl chains, thus supporting the alternating AB packing for space filling reasons. In addition, the larger layer distance and an easier zigzag layer modulation might contribute to *p*2*mm* stabilization in the case of linear side chains.^[^ ^61^ ^]^

### 3.4 Single‐Network Phases

Remarkably, the phase range of the DG is limited to *L*_C_ = 14–18 (*V*_C_ = 30–38) for compounds **K** **/** ***n*** and even to only *L*_C_ = 16–18 (*V*_C_ = 32–36) for compounds ***π*** **/** ***n***. The transition Cub_net_/*Ia* $\bar{3}$ *d* (DG) to Cub_net_/*Fd* $\bar{3}$ *m* (SD) takes place for *L*_C_ = 20 (*V*_C_ = 42) and *L*_C_ = 18–20 (*V*_C_ = 36–40) in series **K** **/** ***n*** ^32^ and ***π*** **/** ***n***, respectively (Figure 8b,c). For the series of **π/** ***n*** compounds with larger side‐chain volumes (*V*_C_ > 46) it turned out that the Cub_net_/*Fd* $\bar{3}$ *m* (SD) phase is the only stable phase over a wide chain length range from *L*_C_ = 20–30 (*V*_C_ = 40–60) and it breaks down without transition to any other mesophase by further chain expansion to *L*_C_ = 32 (*V*_C_ = 64). As the chain elongation leads to a reduction of the number of terphenyls in the cross section of the rod bundles forming the SD network (Table 1), it appears that there is a minimum size of the polar glycerol domains of at least 26 glycerol units to provide a sufficient number of cohesive hydrogen bonding for LC self‐assembly.^[^ ^91^, ^92^ ^]^ A transition to the SG by reduction of the valence of the nodes in the single‐network structure to only three by further chain expansion, as suggested by Figure 1g, is apparently not possible, because it would further reduce the number of cohesive hydrogen bonding in the polar domains.

The DG and SD network phases are distinct from the usual bicontinuous cubic phases, because there is only one continuum formed by the alkyl side chains, while the networks are not continuous, but nanosegregated into polar spheres and *p*‐terphenyls bundles. Thus, these segmented network phases of the triblock BPs **K** **/** ***n*** and ***π*** **/** ***n*** can be regarded as sphere packings,^[^ ^93^, ^94^, ^95^, ^96^, ^97^, ^98^, ^99^, ^100^, ^101^, ^102^, ^103^, ^104^, ^105^ ^]^ too, where the polar “spheres” can be considered as “superatoms” or “mesoatoms”^[^ ^106^, ^107^, ^108^, ^109^, ^110^ ^]^ with distinct valence (*ν*) at the junctions which are interconnected by struts of *p*‐terphenyl bundles forming the “bonds.”^[^ ^111^ ^]^ There are two types of unicontinuous networks for the K‐ and *π*‐shaped molecules, the DG with trigonal 3‐way junctions and the SD with tetrahedral four‐way junctions. The DG is found only over a short range of the side‐chain length (*L*_C_ = 14–18 for **K** **/** ***n*** and 16–18 for ***π*** **/** ***n***) and it is in both series accompanied by a Lam_Sm_ phase at low temperature. In contrast, the SD is stable over a wide chain length range (*L*_C_ = 20–30) and is the only LC phase observed in the whole mesomorphic temperature range of these compounds (Figure 8b,c). Thus, the DG, which is the most common network phase in low‐ and high‐molecular‐mass (block copolymers) amphiphilic systems,^[^ ^4^, ^6^, ^7^, ^11^ ^]^ might be considered here as an intermediate phase at the honeycomb‐to‐SD transition. One contribution stabilizing the D‐ over the G‐network might arise from the fact that the valence has an effect on the precise shape of the polar domains, thus deviating from being exactly spherical. The change of the shape of the polar domains from being triangular planar in G^[^ ^112^ ^]^ to tetrahedral in D^[^ ^23^, ^113^ ^]^ reduces the surface area of the polar glycerol domains and removes any tendency of layer formation induced by the parallel alignment of the planar (flattened)^[^ ^112^ ^]^ three‐way junctions. A second contribution arises from the possibility to transform the strongly elliptical rod bundles (ribbons) in the DG (*n*_bundle_ = 12) to more circular in SD (*n*_bundle_ = 6.6–9) by reduction of the number of molecules organized in their cross section, while retaining the number of glycerols organized in each of the polar domains (by increasing the valence from 3 to 4). This reduces the interfacial area between glycerols and alkyl chain continuum and further increases the degree of negative IMDS^R^ curvature. These effects all together (see Figure 1) might represent the driving force behind the DG to SD transition and the unexpected stability of the D‐net in these soft matter systems. In the DD phase^[^ ^65^, ^66^ ^]^ the number of struts per constant volume unit would be larger than in the DG and this requires the disintegration of the double to the single (SD) network at the G to D transition during chain volume expansion. While the interpenetration of two networks is required for the G‐type network to optimize space filling, their disintegration is favored with growing chain volume and growing valence of the junctions. It appears that the D‐network (*ν* = 4) is the first one being capable of forming a double (DD) as well as a single network (SD),^[^ ^31^, ^32^ ^]^ while the gyroid (*ν* = 3) requires interpenetration to be stable in soft matter systems. Also, the Plumber's Nightmare network with six‐way junctions (*ν* = 6) is known as a single‐ (SP)^[^ ^67^ ^]^ and a double‐network structure (DP,^[^ ^114^ ^]^ see Figure 1c,f), while further increasing the valence of the junctions (*ν*) favors the single networks with respect to the double networks, as, for example, shown for the I‐WP (*ν* = 8)^[^ ^67^ ^]^ and the A15 networks (*ν* = 12,14).^[^ ^68^ ^]^ Thus, with growing mesoatom valence there is growing driving force for single‐network formation.

It is concluded, that for unicontinuous network phases the single diamond with four‐way junctions is the first LC phase capable of forming a single network, while this is obviously not possible for the gyroid with three‐way junctions. Though there are recent simulations suggesting the possibility of existence of the SG in specifically designed block copolymers,^[^ ^14^, ^15^, ^16^, ^17^, ^18^, ^19^ ^]^ it remains to be tested with real low molecular weight and polymeric amphiphilic and polyphilic block molecules if the proposed instability of the DG is only valid for the special case of the of rigid triblock BPs or it can be generalized for other soft matter systems.

## 4 Conclusion

**Figure 10:**
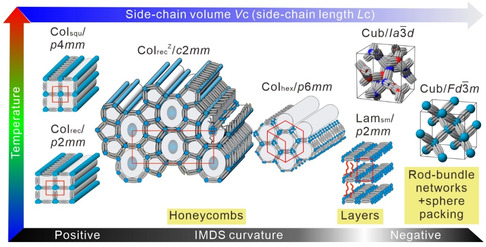
Overall phase sequence of the *π*‐shaped BPs ***π*** **/** ***n*** depending on the side‐chain length and temperature.

The complete series of new catechol‐based BP molecules, having two adjacent linear chains located at the same side (*π*‐shaped BPs) of a *p*‐terphenyl platform and side‐chain lengths ranging from *n* = 6 to *n* = 32, was synthesized and investigated. A sequence of seven LC phases, involving square and rectangular honeycombs, a complex tiling by pentagons, and octagons with the octagons filled by additional columns of coaxial molecular bundles via a hexagonal honeycomb and a correlated lamellar phase to a DG and finally to a SD phase was observed (**Figure** **10**). This series is very distinct from that found for related T‐shaped molecules with only a single‐linear chain (Figure 8a) which exclusively forms monohedral honeycombs^[^ ^75^ ^]^ and it is similar to the K‐shaped compounds with a single but branched side chain (see Figure 8c).^[^ ^32^ ^]^

A unique feature of the *π*‐shaped compounds is the formation of a wide range of the thermodynamically stable single‐network cubic phase with tetrahedral four‐way junctions, the SD, being predicted,^[^ ^14^, ^37^ ^]^ but not yet observed in any other self‐assembling soft matter systems, including lyotropics and block copolymers. It turned out that there is a surprisingly broad existence range of this SD phase with respect to side‐chain volume (*n* = 20–30) and temperature range. Moreover, once formed the SD phase removes all other competing LC phases. However, after exceeding a certain side‐chain volume, and hence reaching a lower critical size of the glycerol spheres at the junctions of the SD nets the capability of network formation is lost for *n* = 32 without transition to the initially expected SG network, having a smaller valence of the “mesoatoms” at the junctions. It appears that in soft‐matter systems the gyroid with three‐way junctions can only be thermodynamically stable if it can interpenetrate with formation of the DG. The diamond net with four‐way junctions is the network with smallest valence being also stable as a single network. This observation might fertilize the search for thermodynamically stable single‐network phases formed in a bottom‐up approach by spontaneous self‐assembly in other systems, like aqueous lyotropics, sol–gel systems, giant amphiphiles,^[^ ^99^ ^]^ and block copolymers,^[^ ^100^ ^]^ which would be of significant interest for applications in photonic materials.^[^ ^70^ ^]^

More generally, it is shown that the precise fine tuning of the structure of small organic molecules, in this case based on the simple *p*‐terphenyl scaffold, allows the tailoring of a large number of very different and complex modes of soft self‐assembly at the low nanometer scale. These highly dynamic systems show structural relations to solid‐state nanostructures with fixed bonds, as for example the zeolitic and network structures of aluminosilicates,^[^ ^115^ ^]^ and the metal–organic and covalent organic solid‐state framework structures produced by reticular chemistry.^[^ ^116^, ^117^, ^118^ ^]^

## 5 Experimental Section

5.1

5.1.1

##### Synthesis

The detailed synthetic procedures and analytical data of the compounds and intermediates are collated in Section S3, Supporting Information.

##### Methods

The obtained compounds ***π*** **/** ***n*** were investigated by POM, DSC, and powder X‐ray diffraction (XRD, SAXS, and WAXS); the details of the used methods, equipment, and conditions are given in the Supporting Information, too (Section S1).

## Conflict of Interest

The authors declare no conflict of interest.

## Author Contributions

**Silvio Poppe**: investigation (lead); writing—original draft (lead). **Changlong Chen**: investigation (equal); methodology (equal). **Yu Cao**: formal analysis (lead); investigation (equal); methodology (equal); supervision (equal); validation (equal); visualization (equal); writing—review and editing (equal). **Feng Liu**: funding acquisition (equal); writing—review and editing (equal). **Carsten Tschierske**: conceptualization (lead); funding acquisition (equal); project administration (lead); supervision (equal); visualization (equal); writing—review and editing (lead).

## Supporting information

Supplementary Material

## Data Availability

The data that support the findings of this study are available in the supplementary material of this article.
