# Supplementary material for: From Columns to Networks: Search toward the Elusive Single Gyroid with π‐Shaped Polyphilic Liquid Crystalline Block Molecules
Source: Small Sci. 2025 May 22;5(7):2500157. doi: 10.1002/smsc.202500157 (PMC12257908; doi:10.1002/smsc.202500157)
Supplement: Supplementary file 1 — Supplementary Material [file SMSC-5-2500157-s001.pdf]

Supporting Information

From columns to networks – Search towards the elusive Single Gyroid with π-shaped polyphilic liquid crystalline block molecules

Silvio Poppe,[a] Changlong Chen,[b] Yu Cao,[b*] Feng Liu,[b] Carsten Tschierske[a*]

[a] Institute of Chemistry, Martin-Luther-University Halle-Wittenberg, Kurt-Mothes-Straße 2, 06120 Halle
[b] State Key Laboratory for Mechanical Behaviour of Materials, Xi'an Jiaotong University, Xi'an 710049 (P.R. China)

Contents

S1. Methods…………………………………………………………………… S3
S2. Additional Data…………………………………………………………… S4
S2.1 DSC traces………………………………………………………………… S4
S2.2 Additional textures……………………………………………………… S6
S2.3 Additional XRD and structural data…………………………………....S7
S2.4 Additional discussion …………………………………………………… S12
S3. Synthesis and Analytical Data ………………………………………… S14
S3.1 General …………………………………………………………………… S14
S3.2 Synthesis of intermediates …………………………………………….. S14
S3.3 Compounds ***π*/*n*** ………………………………………………………… S20
S3.4. Representative NMR spectra ………………………………………… S24
S4. References and Footnotes ……………………………………………… S27

S1. Methods

S1.1 Optical and calorimetric investigations. Phase transitions were determined by polarizing microscopy (Leica DMR XP) in conjunction with a heating stage (FP 82 HT, Mettler) and controller (FP 90, Mettler) and by differential scanning calorimetry (DSC-7, Perkin Elmer) at heating/cooling rates of 10 K min^{-1} (peak temperatures). Optical investigation was carried out under equilibrium conditions between glass slides which were used without further treatment, sample thickness was ~15 µm. A full wavelength retardation plate was used to determine the sign of birefringence (Fig. S2).

S1.2 X-ray scattering. X-ray investigations (Kristalloflex 760H, Siemens) were carried out using Ni filtered CuKα radiation (15 to 30 min exposure time). Aligned samples were obtained on a glass plate. Alignment was achieved upon slow cooling (rate: 1 K min^{-1} – 0.01 K min^{-1}) of a small droplet of the sample. The aligned samples were held on a temperature-controlled heating stage and the diffraction patterns were recorded with a 2D detector (Vantec 500, Bruker). The sample-detector distance for the samples was 9.00 cm for WAXD and 26.70 cm for SAXD measurements.

S1.3 Synchrotron SAXS. High-resolution small-angle and wide-angle powder diffraction experiments were recorded on Beamline BL16B1 at the Shanghai Synchrotron Radiation Facility (SSRF) with beam energy of 12keV and X-ray wavelength of 0.103 nm. Samples were held in evacuated 1 mm capillaries. A modified Linkam hot stage with thermal stability within 0.2 ºC was used, with a hole for the capillary drilled through the silver heating block and mica windows attached to it on each side. A Pilatus 2M detector was used. q Calibration and linearization were verified using several orders of layer reflections from silver behenate and a series of n-alkanes. Experimental diffractograms are fitted using Gaussian-shaped peaks to determine the positions and intensities of the diffraction peaks. The diffraction peaks are indexed based on their peak positions, and the lattice parameters and the space groups are subsequently determined. Once the diffraction intensities are measured, and the corresponding plane group determined, 3D electron density maps can be reconstructed based on the general formula.

$$\rho(xyz) = \sum_{hk} F(hkl)\, e^{[i2\pi(hx+ky+lz)]} \quad (1)$$

Here $F(hkl)$ is the structure factor of a diffraction peak with index (hkl). It is normally a complex number, and the experimentally observed diffraction intensity is

$$I(hkl) = K \cdot F(hkl) = K \cdot |F(hkl)|^2 \quad (2)$$

Here K is a constant related to the sample volume, incident beam intensity etc. If the constant is equal to 1, then the electron density is

$$\rho(xyz) = \sum_{hkl} \sqrt{I(hkl)}\, e^{[i2\pi(hx+ky+lz)+\varphi_{hkl}]} \quad (3)$$

As the observed diffraction intensity $I(hkl)$ is only related to the amplitude of the structure factor $|F(hkl)|$, the information about the phase of $F(hkl)$, Φhkl, cannot be determined directly from the experiment. However, the problem is much simplified when the structure of the ordered phase is centrosymmetric; hence, the structure factor $F(hkl)$ is always real, and Φhk is either 0 or π. The

case is essentially the same for the 2D centrosymmetric phase. This makes it possible for a trial-and-error approach, where candidate electron density maps are reconstructed for all possible phase combinations. The "correct" phase combination is then selected on the merit of the maps, helped by prior physical and chemical knowledge of the system. This is especially useful for studying nanostructures, where typically only a limited number of diffraction peaks are observed.

S2. Additional Data

S2.1 DSC traces

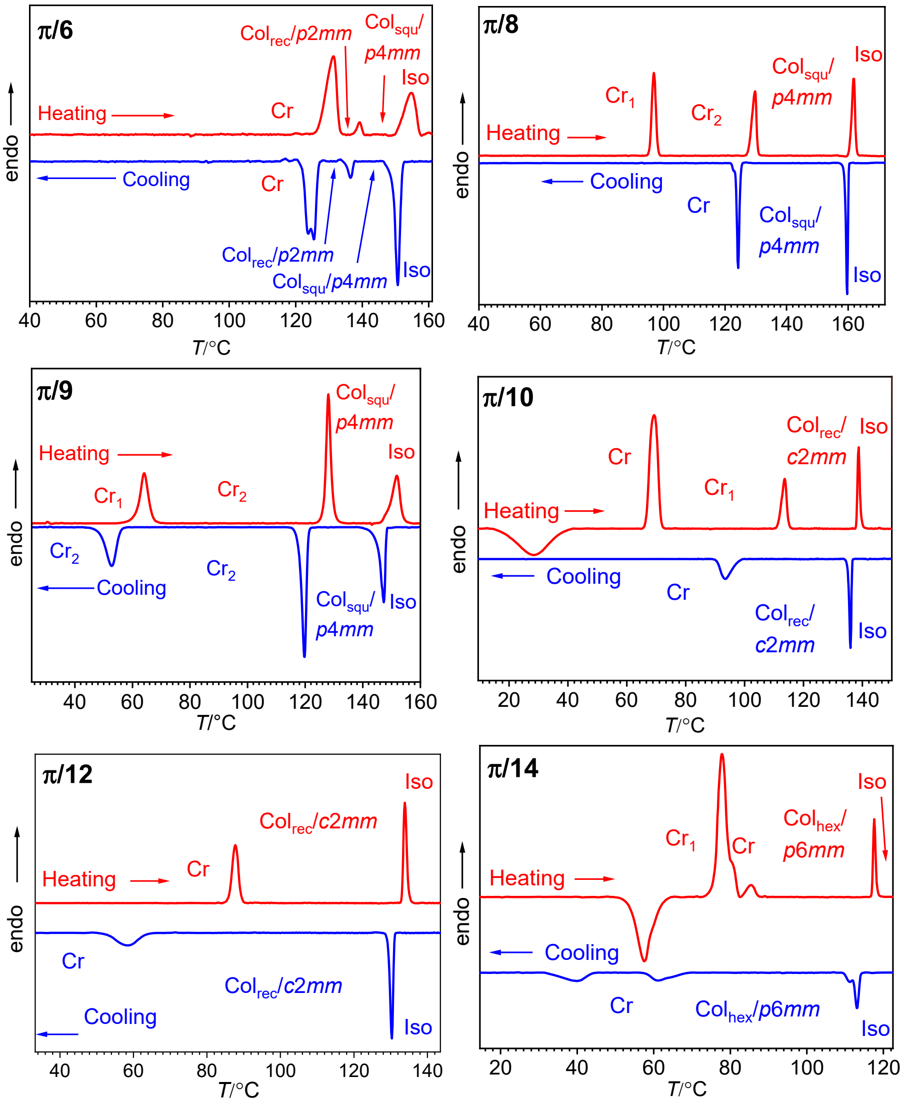

Figure S1: DSC traces of compounds ***π/n*** (second heating and cooling scans, 10 K min^{-1}.

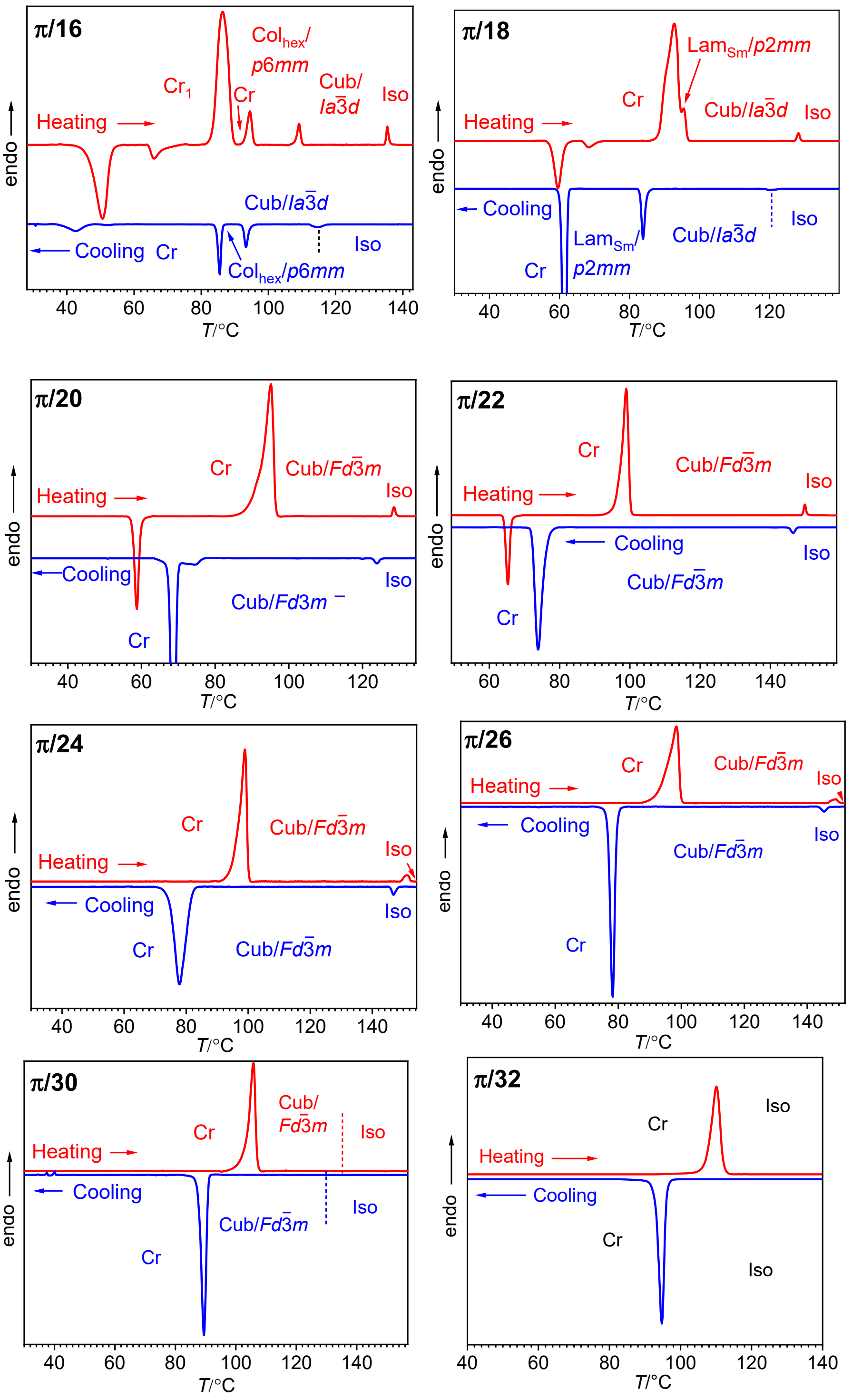

Figure S1 (cont.) DSC traces of compounds **π/*n*** (second heating and cooling scans, 10 K min^{-1}.

S2.2 Additional textures

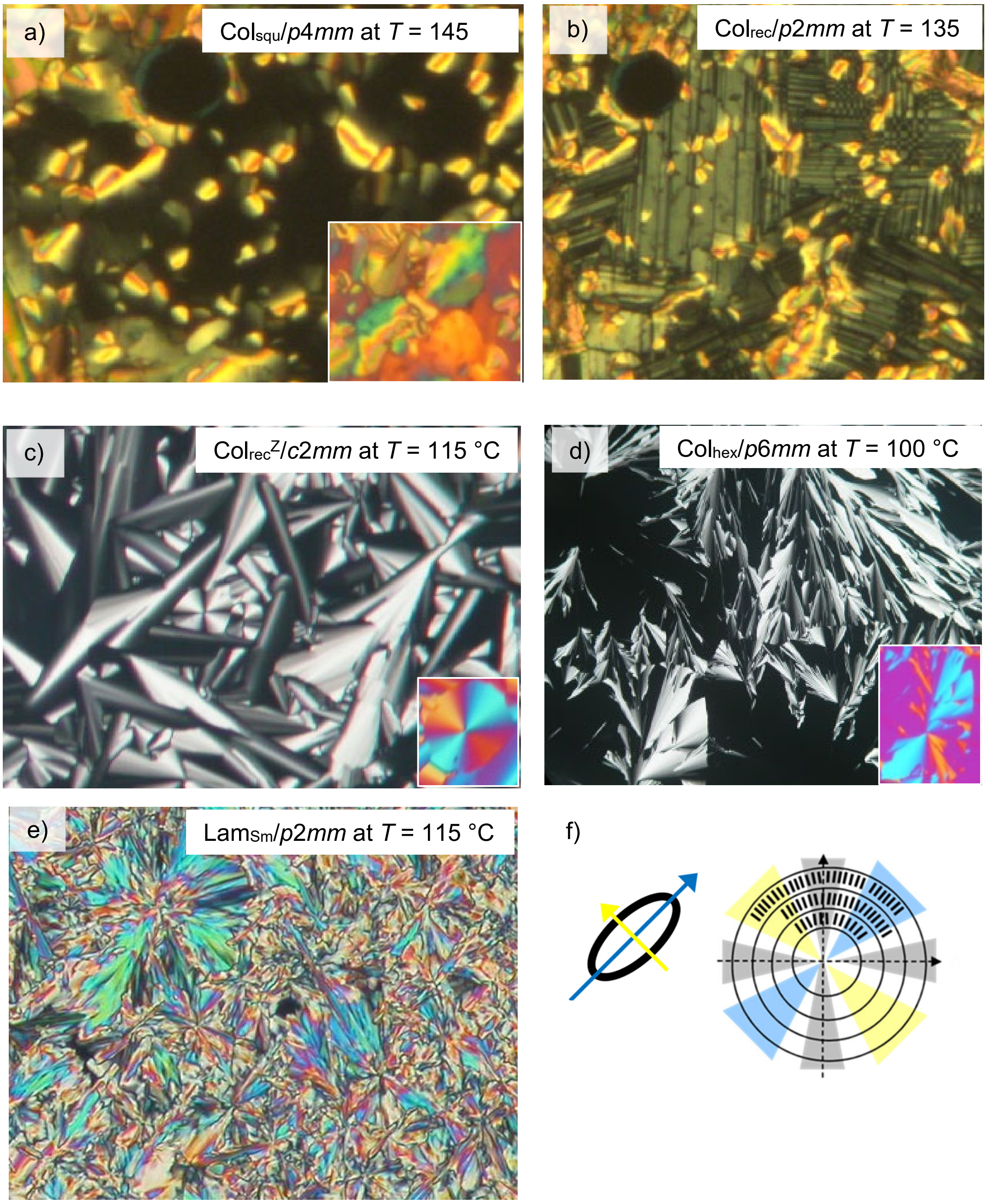

Figure S2. Textures between crossed polarizers of LC phases of **π/*n*** a) $Col_{squ}/p4mm$ phase of **π/6** at T = 145 °C, b) $Col_{rec}/p2mm$ phase of **π/6** at T = 135 °C, c) $Col_{rec}^{Z}/c2mm$ phase of **π/10** at T = 115 °C, d) $Col_{hex}/p6mm$ phase of **π/14** at T = 100 °C, e) Lam/*p*2*mm* phase of **π/18** at T = 80 °C, as inset textures with full wave retarder plate, f) shows the orientation of the indicatrix of the λ-plate and the arrangement of the *p*-terphenyl rods in the spherulitic domains of the columnar honeycomb phases, shown in a-d).

S2.3 Additional XRD and structural data

Table S2. SAXS data of the $Col_{squ}/p4mm$ and $Col_{rec}/p2mm$ phases of **π/6 - π/9.**

Compd.	LC phase	(hkl)	$d_{obs.}$ (nm)	$d_{cal.}$ (nm)	$d_{obs.} - d_{calc}$	intensity	phase	a, b (T/°C)
**π/6**	*p*4*mm*	(10)	2.55	2.55	0.00	100	π	2.55 nm (145 °C)
		(11)	1.80	1.80	0.00	21.7	π	
		(20)	1.28	1.28	0.00	0.9	0	
**π/6**	*p*2*mm*	(01)	2.90	2.90	0.00	100	π	2.33 nm, 2.90 nm (135 °C)
		(10)	2.33	2.33	0.00	37.4	π	
		(11)	1.82	1.82	0.01	16.3	π	
		(02)	1.45	1.45	0.00	7.1	0	
**π/8**	*p*4*mm*	(10)	2.57	2.57	0.00			2.57 nm (140 °C)
		(11)	1.82	1.82	0.00			
		(20)	1.29	1.29	0.00			
**π/9**	*p*4*mm*	(10)	2.58	2.58	0.00	100	π	2.58 nm (145 °C)
		(11)	1.82	1.82	0.01	17.4	π	
		(20)	1.29	1.29	0.01	2.0	0	

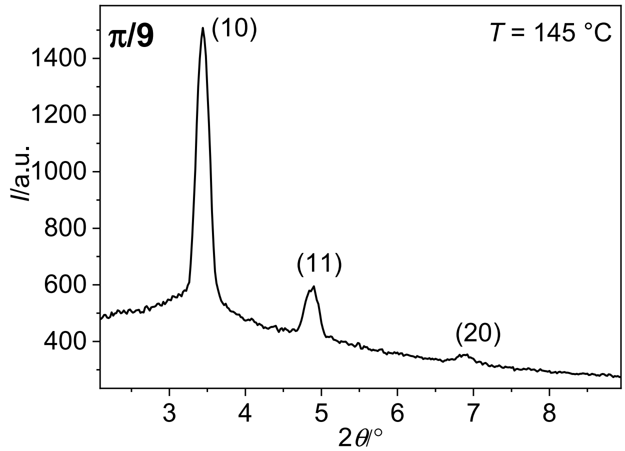

Figure S3. SAXS scan of $Col_{squ}/p4mm$ phase of **π/9** at T = 145 °C.

Table S3: Structural data of the $Col_{squ}/p4mm$ and $Col_{rec}/p2mm$ phases of **π/6 - π/9**. [a]

**Compd.**	**π/6**	**π/6**	**π/8**	**π/9**
LC phase	*p*4*mm*	*p*2*mm*	*p*4*mm*	*p*4*mm*
a,b / nm	2.55	2.33, 2.90	2.57	2.58
L_{mol} / nm	2.60	2.60	2.60	2.60
V_{mol} /nm^3	0.84	0.84	0.94	0.99
V_{cell} / nm^3	2.93	3.04	2.97	2.99
n_{cr}	3.48	3.62	3.16	3.03
n_{liq}	2.73	2.84	2.48	2.38
n_{cell}	3.11	3.23	2.82	2.70
n_{wall}	1.55	1.62	1.41	1.35

[a] Abbreviations: L_{mol} – molecular length, V_{mol} – volume of molecule (determined from the increment system of Immirzi[S1]), V_{cell} – volume of unit cell, n_{cr} – number of molecules in crystalline state, n_{liq} – number of molecules in liquid state, n_{cell} – number of molecules in liquid crystalline state,[S2] n_{wall} – number of molecules per wall.

Table S4. SAXS data of the $Col_{rec}^{Z}/c2mm$ phase compound **π/10**.

Compd.	(*hkl*)	$d_{obs.}$ (nm)	$d_{cal.}$ (nm)	$d_{obs.} - d_{calc}$	*a*, *b* (*T*/°C)
**π/10**	(31)	3.89	3.93	0.04	15.31, 6.16 nm (130 °C)
	(40)	3.83	3.83	0.00	
	(02)	3.08	3.08	0.00	
	(22)	2.85	2.86	0.00	
	(51)	2.74	2.74	0.01	
	(60)	2.56	2.55	0.01	
	(71)	2.05	2.06	0.01	
	(80)	1.92	1.91	0.01	
	(91)	1.65	1.64	0.01	
	(12 0)	1.29	1.28	0.01	

Table S5. Experimental and calculated *d*-spacings, relative integrated intensities, and phases used in the reconstruction of electron densities for the $Col_{rec}^{Z}/c2mm$ phase of **π/12** at 119 ºC. All intensities values are Lorentz and multiplicity corrected.

(*hk*)	$d_{obs.}$- spacings (nm)	$d_{cal.}$- spacings (nm)	Intensity	Phase
(2 0)	7.86	7.86	0.2	0
(1 1)	5.83	5.83	0.2	π
(3 1)	4.02	4.02	13.3	π
(4 0)	3.93	3.93	27.3	0
(0 2)	3.13	3.14	1.7	π
(2 2)	2.91	2.92	100.0	0
(5 1)	2.81	2.81	79.6	0
(6 0)	2.62	2.62	31.5	0
(4 2)	2.45	2.45	2.3	/
(7 1)	2.11	2.11	2.6	/
(1 3)	2.07	2.08	0.2	/
(6 2)	2.01	2.01	7.1	/
(8 0)	1.96	1.97	16.9	/
(3 3)	1.94	1.94	10.1	/
(5 3)	1.74	1.74	6.5	/
(9 1)	1.68	1.68	6.5	/
(8 2)	1.66	1.67	3.4	/
(0 4)	1.57	1.57	3.0	/
(2 4)	1.54	1.54	1.0	/
(7 3)	1.53	1.53	1.2	/
(4 4)	1.45	1.46	0.5	/
(11 1)	1.40	1.39	0.2	/
(9 3)	1.34	1.34	0.8	/
(6 4)	1.34	1.35	1.7	
(12 0)	1.31	1.31	1.8	
(8 4)	1.22	1.23	2.5	/
(3 5)	1.22	1.22	1.1	/
(13 1)	1.18	1.19	1.0	/
(11 3)	1.18	1.18	1.7	/
(5 5)	1.16	1.17	1.6	/
(7 5)	1.09	1.10	0.1	/
(0 6)	1.05	1.05	0.2	/
(2 6)	1.03	1.04	0.1	/
(4 6)	1.00	1.01	0.2	/
(6 6)	0.98	0.97	0.2	/
a_{rec} = 15.72 nm b_{rec} = 6.28 nm				

Table S6: Structural data of the $\mathrm{Col_{rec}}^{Z}/c2mm$ phase of **π/10** and **π/12**. [a]

**Compd.**	**π/10**	**π/12**
LC phase	*c2mm*	*c2mm*
a_{rec} / nm	15.31	15.72
b_{rec} / nm	6.16	6.28
L_{mol} / nm	2.60	2.60
V_{mol} /nm^3	1.04	1.14
V_{cell} / nm^3	42.44	44.42
n_{cr}	40.81	38.97
n_{liq}	32.06	30.62
n_{cell}	36.44	34.79
n_{wall}/n_{bundle}	n.d.	1.73/9.32[b]

[a] Abbreviations: see Table S3, n_{bundle} – number of molecules in the cross section of the rod-bundle columns filling the octagonal cells.[b] Estimation of n_{bundle} and n_{wall} is based on the measurement from reconstructed ED map. The high ED area of the two bundles in each unit cell occupies 4.903 nm^2 for **π/12**. Assuming the molecules as cylinder and length of them along bundle is 2.25 nm, volume is 0.592 nm^3 in the LC state, the average area each molecule occupied would be 0.263 nm^2. This leads to 18.64 molecules in the bundles, which means n_{bundle} as 9.32. Assuming the honeycomb height as 0.45 nm, these bundles contribute with 3.72 molecules per unit cell, reducing n_{cell} to 31.07 for **π/12**. As there are 18 walls per unit cell, n_{wall} can be derived as 1.73. The values for **π/10** should be close to these values considering similar n_{cell}.

Table S7. SAXS data of mesophases of compounds **π/14 – π/18**.

Compd.	LC phase	(*hkl*)	$d_{obs.}$ (nm)	$d_{cal.}$ (nm)	$d_{obs.} - d_{calc}$	intensity	phase	*a*, *b* (*T*/°C)
**π/14**	*p6mm*	(10)	3.59	3.59	0.00			4.15 nm (100 °C)
		(20)	1.80	1.80	0.01			
		(21)	1.37	1.36	0.01			
**π/16**	*Ia*3*d*	(211)	3.12	3.12	0.00	100.0	π	7.64 nm (105 °C)
		(220)	2.70	2.70	0.00	11.5	π	
		(321)	2.04	2.04	0.00	0.7	0	
		(400)	1.91	1.91	0.00	10.2	π	
		(420)	1.71	1.71	0.00	2.9	π	
		(332)	1.63	1.63	0.00	0.3	0	
		(422)	1.56	1.56	0.00	1.8	0	
		(431)	1.50	1.50	0.00	0.3	0	
		(440)	1.35	1.35	0.00	2.1	0	
		(532)	1.24	1.24	0.00	0.03	-	
		(541)	1.18	1.18	0.00	0.2	-	
		(631)	1.12	1.13	0.01	0.3	-	
		(444)	1.10	1.10	0.00	0.7	-	
		(543)	1.08	1.08	0.00	0.1	-	
**π/16**	*p6mm*	(10)	3.64	3.64	0.00	100.0	π	4.20 nm (95 °C)
		(11)	2.10	2.10	0.00	0.7	π	
		(20)	1.82	1.82	0.00	14.3	π	
		(21)	1.38	1.37	0.01	2.6	π	
		(30)	1.21	1.21	0.00	0.6	π	
**π/18**	*Ia*3*d*	(211)	3.12	3.12	0.00	100.0	π	7.76 nm (100 °C)
		(220)	2.70	2.70	0.00	5.9	π	
		(321)	2.04	2.04	0.00	0.7	0	
		(400)	1.91	1.91	0.00	9.3	π	
		(420)	1.71	1.71	0.00	4.5	π	
		(332)	1.63	1.63	0.00	0.8	0	
		(422)	1.56	1.56	0.00	3.4	0	
		(431)	1.50	1.50	0.00	0.3	0	
		(440)	1.35	1.35	0.00	1.6	0	
**π/18**	*p2mm*	(10)	3.27	3.27	0.00	100.0	π	2.36, 3.27 nm (75 °C)
		(01)	2.36	2.36	0.00	1.3	π	
		(20)	1.64	1.64	0.00	10.6	0	

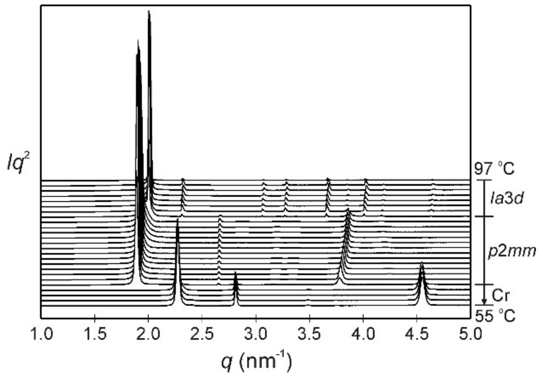

Figure S4. Waterfall diagram of SAXS scans between $T = 55$ °C and $T = 97$ °C of compound **π/18**.

Table S8: Structural data of the mesophases of **π/14 - π/18**. [a]

**Compd.**	**π/14**	**π/16**	**π/16**	**π/18**	**π/18**
LC phase	*p6mm*	*p6mm*	*Ia3d*	*p2mm*	*Ia3d*
a,b / nm	4.15	4.20	7.64	3.27, 2.36	7.76
L_{mol} / nm	2.60	2.60	2.60	2.60	2.60
V_{mol} /nm^3	1.24	1.34	1.34	1.44	1.44
V_{cell} / nm^3	6.86	7.03	446	3.47	467
n_{cr}	5.53	5.24	333	2.41	324
n_{liq}	4.35	4.12	261	1.89	255
n_{cell}	4.94	4.68	297	2.15	290
n_{wall}/n_{bundle}	1.65	1.56	12.4	2.15	12.1
d_{knots}/nm			2.70		2.74

[a] Abbreviations: see Table S3.

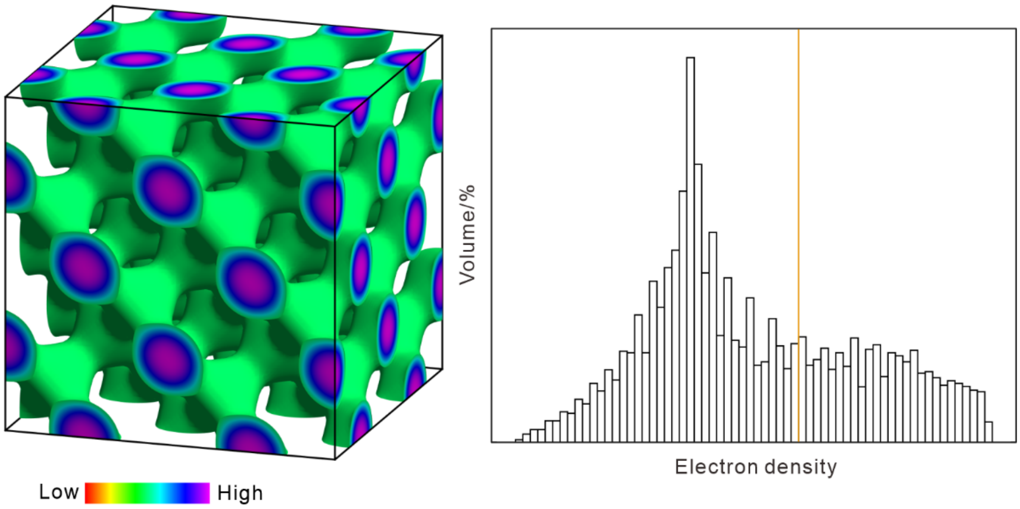

Figure S5. 2x2x2 Reconstructed ED map and histogram of $Fd\bar{3}m$ phase with all seven peaks in Table S9 to highlight the four-way junctions.

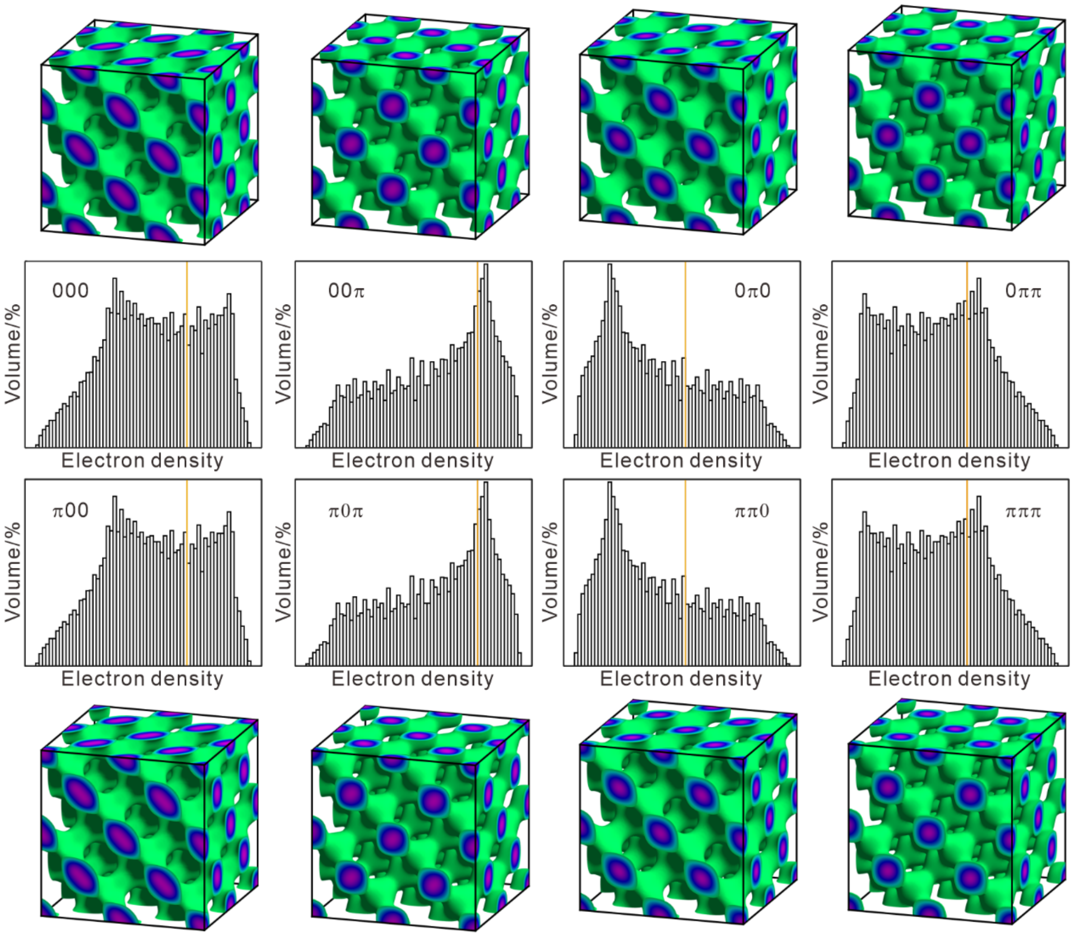

Figure S6. 2x2x2 Reconstructed ED maps and related electron density distribution histograms of $Fd\bar{3}m$ phase with three strongest peaks (111), (220) and (222).

The proper reconstructed ED map can be selected by trial and error with all phase combinations. The low electron density region, which is not shown for clarity, is composed by soft alkyl chains. In liquid crystal state, alkyl chains are flexible enough to show most thermodynamically stable state. In this way, the low electron density region is supposed to exhibit a sharp distribution as majority alkyl chains are in the same state with relatively uniform electron density, either too low or too high. It's clear that 0π0 and ππ0 phase combinations are good choice from histogram. The two choices are essentially same except for an overall shift along (111) direction.

Table S9. SAXS data of the Cub/*Fd*3*m* phase of compounds **π/20** – **π/30**.

Compd.	(hkl)	$d_{obs.}$ (nm)	$d_{cal.}$ (nm)	$d_{obs.} - d_{calc}$	intensity	phase	a (T/°C)
**π/20**	(111)	3.64	3.64	0.00	100.0	0	6.30 nm (100 °C)
	(220)	2.23	2.23	0.00	1.0	π	
	(311)	1.90	1.90	0.00	0.6	0	
	(222)	1.82	1.82	0.00	3.0	0	
	(400)	1.58	1.58	0.00	0.5	0	
	(331)	1.45	1.45	0.00	0.1	0	
	(422)	1.29	1.29	0.00	0.1	π	
**π/22**	(111)	3.63	3.63	0.00	-	-	6.29 nm (120 °C)
	(220)	2.19	2.22	0.03	-	-	
	(400)	1.61	1.58	0.03	-	-	
	(440)	1.10	1.11	0.01	-	-	
**π/24**	(111)	3.59	3.59	0.00	-	-	6.21 nm (130 °C)
	(220)	2.21	2.20	0.01	-	-	
	(311)	1.88	1.87	0.01	-	-	
	(222)	1.80	1.79	0.01	-	-	
**π/26**	(111)	3.594	3.594	0.00	-	-	6.22 nm (120 °C)
	(220)	2.208	2.201	0.01	-	-	
	(222)	1.802	1.797	0.01	-	-	
**π/30**	(111)	3.62	3.62	0.00	-	-	6.22 nm (120 °C)
	(220)	2.23	2.22	0.01	-	-	
	(311)	1.88	1.89	0.01	-	-	
	(222)	1.82	1.81	0.01	-	-	

Table S10: Structural data of the Cub/*Fd*3*m* phase of **π/20** – **π/30**. [a]

**Compd.**	**π/20**	**π/22**	**π/24**	**π/26**	**π/30**
a_{cub} / nm	6.30	6.29	6.21	6.22	6.22
L_{mol} / nm	2.60	2.60	2.60	2.60	2.60
V_{mol} /nm^3	1.54	1.64	1.74	1.84	2.04
V_{cell} / nm^3	250	249	239	241	241
n_{cr}	162	152	137	131	118
n_{liq}	127	119	108	103	93
n_{cell}	145	136	123	117	106
d_{nodes} / nm	2.73	2.72	2.69	2.69	2.69
n_{bundle}	9.06	8.50	7.69	7.31	6.63

[a] Abbreviations: see Table S3; d_{knots} – distance between two junctions, n_{bundle} – number of molecules of one bundle connecting the junctions.

S2.4 Additional Discussion

Details of the *p*4*mm* phase structure. The number of molecules in a unit cell (n_{cell}) with a height of h = 0.45 nm, as calculated by $n_{cell} = 0.893$ (V_{cell}/V_{mol}), where V_{cell} is the unit cell volume, V_{mol} is the molecular volume as determined by Immirzi's increments[S1] and 0.893 is a correction factor for the looser packing in the LC state amounts n_{cell} = 3.1-2.7 molecules, decreasing with growing alkyl chain length (Tables 1, S3). This means that in this square honeycomb each columnar wall is

formed by n_{wall} ~ 1.5 (**π/6**), 1.4 (**π/8**) and 1.3 (**π/9**) molecules in the cross section. This number between n_{wall} = 1 and 2 indicates a staggered packing of the *p*-terphenyls along the honeycomb walls (see Fig. S7b, middle). With increasing chain volume, the lattice parameter a_{squ} (fixed by L_{mol} to narrow limits) remain almost constant while n_{cell} decreases, indicating an expansion along the *c*-axis which effectively leads to the reduction of n_{wall}.

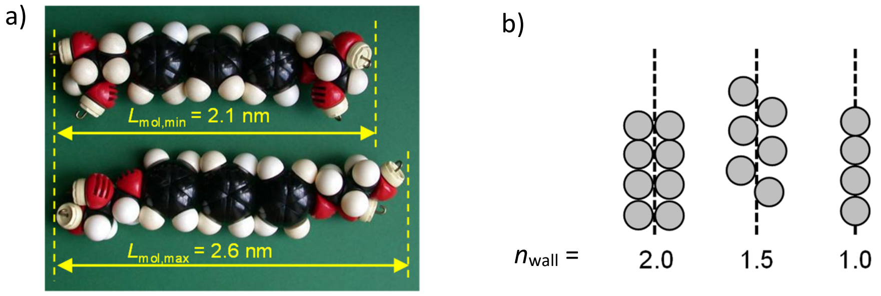

Figure S7. a) CPK models of the rigid core of the compounds ***π/n*** in the conformation with a minimal molecular length of L_{mol} = 2.1 nm (top) and in maximal length with L_{mol} = 2.6 nm (bottom) and b) cross section through a honeycomb wall; the view is along the *p*-terphenyl cores, which are assumed to be rotationally disordered and therefore averaged to a circular cross sectional shape; depending on the number of molecules in the cross-section (n_{wall}), from left to right: n_{wall} = 2.0: back-to-back packed double molecule wall; 1.0 < n_{wall} <2.0: staggered zigzag-like packing of the *p*-terphenyls and n_{wall} = 1.0: single molecule wall.

p*4*mm*-*p*2*mm* transition.** The deformation of the square honeycomb to a rectangular is thought to be due to the higher *trans* fraction of the alkyl side-chains at reduced temperature, though the chains remain in a fluid-like liquid state as confirmed by WAXS (see inset in Figure 3d). This partial chain stiffening supports the parallel alignment of the chains with respect to each other and with respect to two opposite sides of the squares and leads to an elliptical deformation of the alkyl chain domains as well as the glycerol columns. As a result, the square honeycombs change to rectangular with two sides stretched (b_{rec} = 2.90 nm) and two sides shrunk (a_{rec} = 2.33 nm) by approximately the same difference (Col$_{rec}$/*p*2*mm*). While the average number of molecules forming the columnar walls remains almost the same as in the *p*4*mm* phase (see Table S3), their distribution between the walls changes significantly. The ED map in Figure 3d show that the majority of *p*-terphenyls become organized along direction *a*, thus forming layers which are connected by thinner and less well segregated walls along *b*. Thus, the Col$_{rec}$/*p*2*mm* phase is a bit related to the Lam$_{Sm}$/*p*2*mm* phase formed by **π/18** below the $Ia\bar{3}d$ phase (see below), where the walls along *b* are removed. It is also noted that this rectangular honeycomb is distinct from those resulting from a tilt of the molecules in two opposing walls,[3] as obvious from the development of the lattice parameters at the *p*4*mm*-*p*2*mm* transition and the ED maps. Remarkably, the cell area slightly increases from 6.5 to 6.8 nm^2 at the *p*4*mm*-*p*2*mm* transition, which means that obviously the onset of orientational order, and not the thermal chain shrinkage, drives this phase transition. This transition is also observed in the series of compounds ***T/n and ***K/n*** for *n* = 8-12 and *n* = 6, respectively.[S4,S5] For compounds ***π/n*** the *p*4*mm*-*p*2*mm* transition is only found for compound **π/6** (extended side chain length L_R = 0.95

nm in *all-trans* conformation), while the $Col_{squ}/p4mm$ phase is retained (at least down to the crystallization temperature at 120-124 °C) for compounds **π/8** and **π/9** with longer side-chains (L_R = 1.2-1.3 nm in *all-trans* conformation). We attribute this to the fixed size of the square frames with a side length defined by the molecular length (L_{mol} = 2.1 – 2.6 nm). As soon as the length of the side-chains exceed the *p*-terphenyl length (~1.2 nm) the chains have to remain in a more disordered state to avoid the collisions with the honeycomb walls. For these compounds the square cells are thus retained at reduced temperature. Moreover, the longer chains provide an increased space filling in the square cells, additionally inhibiting their deformation to rectangular.

S3. Synthesis and Analytical Data

S3.1 General

Unless otherwise noted, all starting materials were purchased from commercial sources and were used without further purification. 1,4-diiodo-2,3-dihydroxybenzene[S6] and 4-[(2,2-dimethyl-1,3-dioxolane-4-yl)methoxy]benzeneboronic acid (**1**)[S7] was synthesized as reported previously. 1-Bromoalkanes with *n* = 4-22 were commercially available and purchased from Sigma Aldrich. Bromotricontane (*n* = 30) and bromodotricontane (*n* = 32) were prepared from 1-triacontanol and 1-dotricontanol which were purchased from Phygenera and Shanghai Terppon, respectively, with triphenylphosphine and *N*-bromosuccinimide.[S8] The synthesis of the *n*-alkyl bromides with *n* = 24 and 26 is described in ref.[S9]

Column chromatography was performed with silica gel 60 (63-200 µm, Fluka). The purity of all products was checked with thin layer chromatography (silicagel 60 F_{254}, Merck). $CHCl_3$ and $CHCl_3$/MeOH mixtures were used as eluents and the spots were detected by UV radiation. Determination of structures of intermediates and end-products was obtained by NMR spectroscopy (VARIAN Gemini 2000 and Unity Inova 500, all spectra were recorded at 27 °C, with exception of those of **π/32** which were recorded at 55 °C to improve solubility). Microanalyses were performed using a CARLO Erba-CHNO 1102 elemental analyzer and HR-MS was recorded with a micoTOF HR-ESI mass spectrometer (Bruker).

S3.2 Synthesis of intermediates

S3.2.1 Synthesis of 3,6-diiodo-1,2-dialkoxybenzenes (2/*n*)

According to ref. [S6] a mixture of the 3,6-diiodocatechol (1 equ., ~0.7-1.5 mmol), the appropriate 1-bromoalkane (2.5 equ.), K_2CO_3 (5 equ.) and Bu_4NI (tip of a spatula) in anhydrous DMF (30 mL/~0.7 mmol catechol) was stirred at 120 °C for 12 h.[S10] After cooling to room temperature, the reaction was poured into water (50 mL) and the aqueous layer was extracted with Et_2O or with EtOAc (3x50 mL). The combined organic layers were washed with saturated aqu. LiCl, water and brine (50 mL each). After drying over anhydrous Na_2SO_4, filtration and evaporation of the solvent (rotatory evaporator, the crude product was purified by column chromatography and used without further purification for the next step.

3,6-Diiodo-1,2-dihexyloxybenzene 2/6: Synthesized from 3,6-diiodocatechol (250 mg, 0.69 mmol), 1-bromohexane (250 mg, 1.52 mmol) with K_2CO_3 (960 mg, 6.90 mmol) as base and Bu_4NI (10 mg, 0.03 mmol) as catalyst in DMF (30 mL). Purification by column chromatography (eluent: *n*-hexane/$CHCl_3$ = 4/1), colorless oil, $C_{18}H_{28}I_2O_2$, M = 530.22 g/mol, yield: 340 mg (93%), **^{1}H-**

NMR ($CDCl_3$, 400 MHz): δ / ppm = 7.22 (s, 2H, Aryl-*H*), 3.98 (t, $^3J_{H,H}$ = 6.7 Hz, 4H, OCH_2), 1.89 – 1.77 (m, 4H, CH_2), 1.52 – 1.44 (m, 4H, CH_2), 1.41 – 1.21 (m, 8H, CH_2), 0.89 (t, $^3J_{H,H}$ = 6.8 Hz, 6H, CH_3).

3,6-Diiodo-1,2-dioctyloxybenzene 2/8: Synthesized from 3,6-diiodocatechol (250 mg, 0.69 mmol), 1-bromoctane (300 mg, 1.52 mmol), K_2CO_3 (960 mg, 6.90 mmol), Bu_4NI (10 mg, 0.03 mmol) in DMF (30 mL). Purification by column chromatography (eluent: *n*-hexane/$CHCl_3$ = 4/1), colorless oil, yield: 360 mg (89%), **^{1}H-NMR** ($CDCl_3$, 400 MHz): δ / ppm = 7.22 (s, 2H, Aryl-*H*), 3.98 (t, $^3J_{H,H}$ = 6.7 Hz, 4H, OCH_2), 1.89 – 1.77 (m, 4H, CH_2), 1.52 – 1.44 (m, 4H, CH_2), 1.41 – 1.21 (m, 16H, CH_2), 0.89 (t, $^3J_{H,H}$ = 6.8 Hz, 6H, CH_3).

3,6-Diiodo-1,2-dinonyloxybenzene 2/9: Synthesized from 3,6-diiodocatechol (500 mg, 1.38 mmol), 1-bromoctane (630 mg, 3.04 mmol), K_2CO_3 (1.90 g, 13.80 mmol), Bu_4NI (10 mg, 0.03 mmol) in DMF (50 mL). Purification by column chromatography (eluent: *n*-hexane), colorless solid, yield: 800 mg (94%), **^{1}H-NMR** ($CDCl_3$, 400 MHz): δ / ppm = 7.22 (s, 2H, Aryl-*H*), 3.98 (t, $^3J_{H,H}$ = 6.7 Hz, 4H, OCH_2), 1.89 – 1.77 (m, 4H, CH_2), 1.52 – 1.44 (m, 4H, CH_2), 1.41 – 1.21 (m, 20H, CH_2), 0.89 (t, $^3J_{H,H}$ = 6.8 Hz, 6H, CH_3).

3,6-Diiodo-1,2-didecyloxybenzene 2/10: Synthesized from 3,6-diiodocatechol (250 mg, 0.69 mmol), 1-bromodecane (340 mg, 1.52 mmol), K_2CO_3 (960 mg, 6.90 mmol), Bu_4NI (10 mg, 0.03 mmol) in DMF (30 mL). Purification by column chromatography (eluent: *n*-hexane/$CHCl_3$ = 4/1), colorless solid, yield: 390 mg (88%), m. p. 62 °C, **^{1}H-NMR** ($CDCl_3$, 400 MHz): δ / ppm = 7.22 (s, 2H, Aryl-*H*), 3.98 (t, $^3J_{H,H}$ = 6.7 Hz, 4H, OCH_2), 1.89 – 1.77 (m, 4H,CH_2), 1.52 – 1.44 (m, 4H, CH_2), 1.41 – 1.21 (m, 24H, CH_2), 0.89 (t, $^3J_{H,H}$ = 6.8 Hz, 6H, CH_3).

3,6-Diiodo-1,2-didodecyloxybenzene 2/12 [S11]: Synthesized from 3,6-diiodocatechol (250 mg, 0.69 mmol), 1-bromododecane (380 mg, 1.52 mmol), K_2CO_3 (960 mg, 6.90 mmol), Bu_4NI (10 mg, 0.03 mmol) in DMF (30 mL). Purification by column chromatography (eluent: *n*-hexane/$CHCl_3$ = 4/1), colorless solid, yield: 410 mg (85%), m. p. 66 °C, **^{1}H-NMR** ($CDCl_3$, 400 MHz): δ / ppm = 7.22 (s, 2H, Aryl-*H*), 3.98 (t, $^3J_{H,H}$ = 6.7 Hz, 4H, OCH_2), 1.89 – 1.77 (m, 4H, CH_2), 1.52 – 1.44 (m, 4H, CH_2), 1.41 – 1.21 (m, 32H, CH_2), 0.89 (t, $^3J_{H,H}$ = 6.8 Hz, 6H, CH_3).

3,6-Diiodo-1,2-ditetradecyloxybenzene 2/14 [S12]: Synthesized from 3,6-diiodocatechol (250 mg, 0.69 mmol), 1-bromotetradecane (430 mg, 1.52 mmol), K_2CO_3 (960 mg, 6.90 mmol), Bu_4NI (10 mg, 0.03 mmol) in DMF (30 mL). Purification by column chromatography (eluent: *n*-hexane/$CHCl_3$ = 4/1), colorless solid, yield: 320 mg (62%), m. p. 70 °C, **^{1}H-NMR** ($CDCl_3$, 400 MHz): δ / ppm = 7.22 (s, 2H, Aryl-*H*), 3.98 (t, $^3J_{H,H}$ = 6.7 Hz, 4H, OCH_2), 1.89 – 1.77 (m, 4H, CH_2), 1.52 – 1.44 (m, 4H, CH_2), 1.41 – 1.21 (m, 40H, CH_2), 0.89 (t, $^3J_{H,H}$ = 6.8 Hz, 6H, CH_3).

3,6-Diiodo-1,2-dihexadecyloxybenzene 2/16: Synthesized from 3,6-diiodocatechol (250 mg, 0.69 mmol), 1-bromohexadecane (470 mg, 1.52 mmol), K_2CO_3 (960 mg, 6.90 mmol), Bu_4NI (10 mg, 0.03 mmol) in DMF (30 mL). Purification by column chromatography (eluent: *n*-hexane/$CHCl_3$ = 4/1), colorless solid, yield: 440 mg (79%), m. p. 75 °C, **^{1}H-NMR** ($CDCl_3$, 400 MHz): δ / ppm = 7.22 (s, 2H, Aryl-*H*), 3.98 (t, $^3J_{H,H}$ = 6.7 Hz, 4H, OCH_2), 1.89 – 1.77 (m, 4H, CH_2), 1.52 – 1.44 (m, 4H, CH_2), 1.41 – 1.21 (m, 48H, CH_2), 0.89 (t, $^3J_{H,H}$ = 6.8 Hz, 6H, CH_3).

3,6-Diiodo-1,2-dioctadecyloxybenzene 2/18:[S11] Synthesized from 3,6-diiodocatechol (250 mg, 0.69 mmol), 1-bromooctadecane (510 mg, 1.52 mmol), K_2CO_3 (960 mg, 6.90 mmol), Bu_4NI (10 mg, 0.03 mmol) in DMF (30 mL). Purification by column chromatography (eluent: *n*-hexane/$CHCl_3$ = 4/1), colorless solid, yield: 590 mg (95%), m. p. 79 °C, **^{1}H-NMR** ($CDCl_3$, 400 MHz): δ/ppm = 7.22 (s, 2H, Aryl-*H*), 3.98 (t, $^3J_{H,H}$ = 6.7 Hz, 4H, OC*H*$_2$), 1.89 – 1.77 (m, 4H, C*H*$_2$), 1.52 – 1.44 (m, 4H, C*H*$_2$), 1.41 – 1.21 (m, 56H, -C*H*$_2$), 0.89 (t, $^3J_{H,H}$ = 6.8 Hz, 6H, C*H*$_3$).

3,6-Diiodo-1,2-dieicosyloxybenzene 2/20: Synthesized from 3,6-diiodocatechol (250 mg, 0.69 mmol), 1-bromoeicosane (550 mg, 1.52 mmol), K_2CO_3 (960 mg, 6.90 mmol), Bu_4NI (10 mg, 0.03 mmol) in DMF (30 mL). Purification by column chromatography (eluent: *n*-hexane/$CHCl_3$ = 4/1), colorless solid, yield: 230 mg (37%), m. p. 84 °C, **^{1}H-NMR** ($CDCl_3$, 400 MHz): δ / ppm = 7.22 (s, 2H, Aryl-*H*), 3.98 (t, $^3J_{H,H}$ = 6.7 Hz, 4H, OC*H*$_2$), 1.89 – 1.77 (m, 4H, C*H*$_2$), 1.52 – 1.44 (m, 4H, C*H*$_2$), 1.41 – 1.21 (m, 64H, C*H*$_2$), 0.89 (t, $^3J_{H,H}$ = 6.8 Hz, 6H, C*H*$_3$).

3,6-Diiodo-1,2-didocosyloxybenzene 2/22: Synthesized from 3,6-diiodocatechol (250 mg, 0.69 mmol), 1-bromodocosane (600 mg, 1.52 mmol), K_2CO_3 (960 mg, 6.90 mmol), Bu_4NI (10 mg, 0.03 mmol) in DMF (30 mL). Purification by column chromatography (eluent: *n*-hexane/$CHCl_3$ = 4/1), colorless solid, yield: 560 mg (83%), m. p. 86 °C, **^{1}H-NMR** ($CDCl_3$, 400 MHz): δ / ppm = 7.22 (s, 2H, Aryl-*H*), 3.98 (t, $^3J_{H,H}$ = 6.7 Hz, 4H, OC*H*$_2$), 1.89 – 1.77 (m, 4H, C*H*$_2$), 1.52 – 1.44 (m, 4H, C*H*$_2$), 1.41 – 1.21 (m, 72H, C*H*$_2$), 0.89 (t, $^3J_{H,H}$ = 6.8 Hz, 6H, C*H*$_3$).

3,6-Diiodo-1,2-ditetracosyloxybenzene 2/24: Synthesized from 3,6-diiodocatechol (500 mg, 1.38 mmol), 1-bromotetracosane (1.32 g, 3.04 mmol), K_2CO_3 (1.90 g, 13.80 mmol), Bu_4NI (10 mg, 0.03 mmol) in DMF (30 mL). Purification by column chromatography (eluent: *n*-hexane/$CHCl_3$ = 4/1), colorless solid, yield: 310 mg (22%), m. p. 79 °C, **^{1}H-NMR** ($CDCl_3$, 400 MHz): δ / ppm = 7.22 (s, 2H, Aryl-*H*), 3.98 (t, $^3J_{H,H}$ = 6.7 Hz, 4H, OC*H*$_2$), 1.90 – 1.74 (m, 4H, C*H*$_2$), 1.58 – 1.41 (m, 4H, C*H*$_2$), 1.41 – 1.17 (m, 80H, C*H*$_2$), 0.88 (t, $^3J_{H,H}$ = 6.8 Hz, 6H, C*H*$_3$).

3,6-Diiodo-1,2-dihexacosyloxybenzene 2/26: Synthesized from 3,6-diiodocatechol (260 mg, 0.71 mmol), 1-bromohexacosane (700 mg, 1.57 mmol), K_2CO_3 (980 mg, 7.10 mmol), Bu_4NI (10 mg, 0.03 mmol) in DMF (30 mL). Purification by column chromatography (eluent: *n*-hexane/$CHCl_3$ = 4/1), colorless solid, yield: 250 mg (32%), m. p.92 °C, **^{1}H-NMR** ($CDCl_3$, 400 MHz): δ / ppm = 7.22 (s, 2H, Aryl-*H*), 3.98 (t, $^3J_{H,H}$ = 6.7 Hz, 4H , OC*H*$_2$), 1.91 – 1.75 (m, 4H, C*H*$_2$), 1.63 – 1.44 (m, 4H, C*H*$_2$), 1.44 – 1.14 (m, 88H, C*H*$_2$), 0.88 (t, $^3J_{H,H}$ = 6.8 Hz, 6H, C*H*$_3$).

3,6-Diiodo-1,2-ditriacontyloxybenzene 2/30: Synthesized from 3,6-diiodocatechol (500 mg, 1.38 mmol), 1-bromotriacontane (1.52 g, 3.04 mmol), K_2CO_3 (1.90 mg, 13.80 mmol), Bu_4NI (10 mg, 0.03 mmol) in DMF (30 mL), purification by column chromatography (eluent: $CHCl_3$), colorless solid, yield: 1.02 g (61%), m. p. 96 °C, **^{1}H-NMR** ($CDCl_3$, 400 MHz): δ / ppm = 7.22 (s, 2H, Aryl-*H*), 3.98 (t, $^3J_{H,H}$ = 6.7 Hz, 4H , OC*H*$_2$), 1.91 – 1.75 (m, 4H, C*H*$_2$), 1.63 – 1.44 (m, 4H, C*H*$_2$), 1.44 – 1.14 (m, 104H, C*H*$_2$), 0.88 (t, $^3J_{H,H}$ = 6.8 Hz, 6H, C*H*$_3$).

3,6-Diiodo-1,2-didotriacontyloxybenzene 2/32: Synthesized from 3,6-diiodocatechol (640 mg, 1.76 mmol), 1-bromodotriacontane (2.05 g, 3.86 mmol), K_2CO_3 (2.43 mg, 17.60 mmol), Bu_4NI (10 mg, 0.03 mmol) in DMF (100 mL). Purification by recrystallization from $CHCl_3$/MeOH, colorless solid, yield: 1.03 g (59%), m. p. 97 °C, **^{1}H-NMR** ($CDCl_3$, 500 MHz): δ / ppm = 7.22 (s, 2H, Aryl-

H), 3.98 (t, $^3J_{H,H}$ = 6.8 Hz, 4H), 1.89 – 1.76 (m, 4H), 1.55 – 1.42 (m, 4H), 1.41 – 1.13 (m, 112H), 0.88 (t, $^3J_{H,H}$ = 6.9 Hz, 6H).

S3.2.2 Synthesis of the acetonides 3/*n* [S13]

The appropriate 3,6-diiodocatecholdialkylether **2/*n*** (1 equ., 0.2-0.8 mmol) and the benzene boronic acid **1** (~2.5 equ.) were suspended in a mixture of sat. $NaHCO_3$-sol./THF (20 mL/30 mL) and degassed for 1 h. Afterwards [$Pd(PPh_3)_4$] (~40 mg/0.5 mmol) was added and the mixture was stirred for 6 h at reflux. Water (100 mL) was added and the mixture was extracted with EtOAc (3x50mL) and the organic layer was washed with water and brine (50 mL each). The solvent was removed under reduced pressure (rotatory evaporator) and the residue was purified by column chromatography.

2,2′-Di-*n*-hexyloxy-4,4′′-bis[(2,2-dimethyl-1,3-dioxolane-4-yl)methoxy]-*p*-terphenyl 3/6: Synthesized from **2/6** (340 mg, 0.64 mmol), **1** (360 mg, 1.41 mmol), [$Pd(PPh_3)_4$] (40 mg, 0.03 mmol), purification by column chromatography (eluent: $CHCl_3$). Colorless oil, yield: 410 mg (85%), **^{1}H-NMR** ($CDCl_3$, 400 MHz): δ / ppm = 7.53 – 7.48 (m, 4H, Aryl-*H*), 7.08 (s, 2H, Aryl-*H*), 6.98 – 6.93 (m, 4H, Aryl-*H*), 4.51 (quin, $^3J_{H,H}$ = 6.11 Hz, 2H, OC*H*), 4.19 (dd, $^3J_{H,H}$ = 8.4, $^3J_{H,H}$ = 6.4 Hz, 2H, OC*H*2), 4.15 – 4.08 (m, 2H, OC*H*2), 4.02 – 3.88 (m, 4H, OC*H*2), 3.76 (t, $^3J_{H,H}$ = 6.6 Hz, 4H, OC*H*2), 1.58 – 1.49 (m, 4H, C*H*2), 1.48 (s, 6H, OC(C*H*3)2), 1.42 (s, 6H, OC(C*H*3)2), 1.36 – 1.13 (m, 12H, C*H*2), 0.88 (t, $^3J_{H,H}$ = 6.9 Hz, 6H, C*H*3).

2,2′-Di-*n*-octyloxy-4,4′′-bis[(2,2-dimethyl-1,3-dioxolane-4-yl)methoxy]-*p*-terphenyl 3/8: Synthesized from **2/8** (360 mg, 0.61 mmol), **1** (340 mg, 1.34 mmol), [$Pd(PPh_3)_4$] (40 mg, 0.03 mmol), purification by column chromatography (eluent: $CHCl_3$). Colorless oil, yield: 430 mg (90%), **^{1}H-NMR** ($CDCl_3$, 400 MHz): δ / ppm = 7.53 – 7.48 (m, 4H, Aryl-*H*), 7.08 (s, 2H, Aryl-*H*), 6.98 – 6.93 (m, 4H, Aryl-*H*), 4.51 (quin, $^3J_{H,H}$ = 6.11 Hz, 2H, OC*H*), 4.19 (dd, $^3J_{H,H}$ = 8.4, $^3J_{H,H}$ = 6.4 Hz, 2H, OC*H*2), 4.15 – 4.08 (m, 2H, OC*H*2), 4.02 – 3.88 (m, 4H, OC*H*2), 3.76 (t, $^3J_{H,H}$ = 6.6 Hz, 4H, OC*H*2), 1.58 – 1.49 (m, 4H, C*H*2), 1.48 (s, 6H, OC(C*H*3)2), 1.42 (s, 6H, OC(C*H*3)2), 1.36 – 1.13 (m, 20H, C*H*2), 0.88 (t, $^3J_{H,H}$ = 6.9 Hz, 6H, C*H*3).

2,2′-Di-*n*-nonyloxy-4,4′′-bis[(2,2-dimethyl-1,3-dioxolane-4-yl)methoxy]-*p*-terphenyl 3/9: Synthesized from **2/9** (500 mg, 0.81 mmol), **1** (500 mg, 1.79 mmol), [$Pd(PPh_3)_4$] (50 mg, 0.04 mmol), purification by column chromatography (eluent: $CHCl_3$). Colorless oil, yield: 480 mg (76%), **^{1}H-NMR** ($CDCl_3$, 400 MHz): δ / ppm = 7.53 – 7.48 (m, 4H, Aryl-*H*), 7.08 (s, 2H, Aryl-*H*), 6.98 – 6.93 (m, 4H, Aryl-*H*), 4.51 (quin, $^3J_{H,H}$ = 6.11 Hz, 2H, OC*H*), 4.19 (dd, $^3J_{H,H}$ = 8.4, $^3J_{H,H}$ = 6.4 Hz, 2H, OC*H*2), 4.15 – 4.08 (m, 2H, OC*H*2), 4.02 – 3.88 (m, 4H, OC*H*2), 3.76 (t, $^3J_{H,H}$ = 6.6 Hz, 4H, OC*H*2), 1.58 – 1.49 (m, 4H, C*H*2), 1.48 (s, 6H, OC(C*H*3)2), 1.42 (s, 6H, OC(C*H*3)2), 1.36 – 1.13 (m, 20H, C*H*2), 0.88 (t, $^3J_{H,H}$ = 6.9 Hz, 6H, C*H*3).

2,2′-Di-*n*-decyloxy-4,4′′-bis[(2,2-dimethyl-1,3-dioxolane-4-yl)methoxy]-*p*-terphenyl 3/10: Synthesized from **2/10** (390 mg, 0.61 mmol), **1** (380 mg, 1.52 mmol), [$Pd(PPh_3)_4$] (50 mg, 0.03 mmol), purification by column chromatography (eluent: $CHCl_3$). Colorless oil, yield: 210 mg (48%), **^{1}H-NMR** ($CDCl_3$, 400 MHz): δ / ppm = 7.53 – 7.48 (m, 4H, Aryl-*H*), 7.08 (s, 2H, Aryl-*H*), 6.98 – 6.93 (m, 4H, Aryl-*H*), 4.51 (quin, $^3J_{H,H}$ = 6.11 Hz, 2H, OC*H*), 4.19 (dd, $^3J_{H,H}$ = 8.4, $^3J_{H,H}$ = 6.4 Hz, 2H, OC*H*2), 4.15 – 4.08 (m, 2H, OC*H*2), 4.02 – 3.88 (m, 4H, OC*H*2), 3.76 (t, $^3J_{H,H}$ = 6.6

Hz, 4H, OCH_2), 1.58 – 1.49 (m, 4H, CH_2), 1.48 (s, 6H, $OC(CH_3)_2$), 1.42 (s, 6H, $OC(CH_3)_2$), 1.36 – 1.13 (m, 28H, CH_2), 0.88 (t, $^3J_{H,H}$ = 6.9 Hz, 6H, CH_3).

2,2´-Di-*n*-dodecyloxy-4,4´´-bis[(2,2-dimethyl-1,3-dioxolane-4-yl)methoxy]-*p*-terphenyl 3/12: Synthesized from **2/12** (410 mg, 0.59 mmol), **1** (370 mg, 1.47 mmol), [$Pd(PPh_3)_4$] (50 mg, 0.03 mmol), purification by column chromatography (eluent: $CHCl_3$). Colorless oil, yield: 220 mg (48%), **^{1}H-NMR** ($CDCl_3$, 400 MHz): δ / ppm = 7.54 – 7.48 (m, 4H, Aryl-*H*), 7.08 (s, 2H, Aryl-*H*), 6.98 – 6.92 (m, 4H, Aryl-*H*), 4.51 (quin, $^3J_{H,H}$ = 6.0 Hz, 2H, OC*H*), 4.19 (dd, $^3J_{H,H}$ = 8.5, $^3J_{H,H}$ = 6.4 Hz, 2H, OCH_2), 4.16 – 4.08 (m, 2H, OCH_2), 4.04 – 3.90 (m, 4H, OCH_2), 3.76 (t, $^3J_{H,H}$ = 6.5 Hz, 4H, OCH_2), 1.58 – 1.50 (m, 4H, CH_2), 1.48 (s, 6H, $OC(CH_3)_2$), 1.42 (s, 6H, $OC(CH_3)_2$), 1.35 – 1.13 (m, 36H, CH_2), 0.88 (t, $^3J_{H,H}$ = 6.9 Hz, 6H, CH_3).

2,2´-Di-*n*-tetradecyloxy-4,4´´-bis[(2,2-dimethyl-1,3-dioxolane-4-yl)methoxy]-*p*-terphenyl 3/14: Synthesized from **2/14** (320 mg, 0.42 mmol), **1** (270 mg, 1.05 mmol), [$Pd(PPh_3)_4$] (50 mg, 0.03 mmol), purification by column chromatography (eluent: $CHCl_3$). Colorless oil, yield: 300 mg (78%), **^{1}H-NMR** ($CDCl_3$, 400 MHz): δ / ppm = 7.54 – 7.48 (m, 4H, Aryl-*H*), 7.08 (s, 2H, Aryl-*H*), 6.98 – 6.93 (m, 4H, Aryl-*H*), 4.55 – 4.47 (quin, $^3J_{H,H}$ = 6.03 Hz, 2H, OC*H*), 4.19 (dd, $^3J_{H,H}$ = 8.5, $^3J_{H,H}$ = 6.4 Hz, 2H, OCH_2), 4.14 – 4.09 (m, 2H, -OCH_2), 4.02 – 3.89 (m, 4H, OCH_2), 3.76 (t, $^3J_{H,H}$ = 6.6 Hz, 4H, OCH_2), 1.58 – 1.50 (m, 4H, CH_2), 1.48 (s, 6H, $OC(CH_3)_2$), 1.42 (s, 6H, $OC(CH_3)_2$), 1.37 – 1.11 (m, 44H, CH_2), 0.88 (t, $^3J_{H,H}$ = 6.9 Hz, 3H, CH_3).

2,2´-Di-*n*-hexadecyloxy-4,4´´-bis[(2,2-dimethyl-1,3-dioxolane-4-yl)methoxy]-*p*-terphenyl 3/16: Synthesized from **2/16** (440 mg, 0.42 mmol), **1** (350 mg, 1.35 mmol), [$Pd(PPh_3)_4$] (50 mg, 0.03 mmol), purification by column chromatography (eluent: $CHCl_3$). Colorless oil, yield: 390 mg (74%), **^{1}H-NMR** ($CDCl_3$, 400 MHz): δ / ppm = 7.53 – 7.48 (m, 4H, Aryl-*H*), 7.08 (s, 2H, Aryl-*H*), 6.98 – 6.93 (m, 4H, Aryl-*H*), 4.51 (quin, $^3J_{H,H}$ = 6.11 Hz, 2H, OC*H*), 4.19 (dd, $^3J_{H,H}$ = 8.4, $^3J_{H,H}$ = 6.4 Hz, 2H, OCH_2), 4.15 – 4.08 (m, 2H, OCH_2), 4.02 – 3.88 (m, 4H, OCH_2), 3.76 (t, $^3J_{H,H}$ = 6.6 Hz, 4H, OCH_2), 1.58 – 1.49 (m, 4H, CH_2), 1.48 (s, 6H, $OC(CH_3)_2$), 1.42 (s, 6H, $OC(CH_3)_2$), 1.36 – 1.13 (m, 52H, CH_2), 0.88 (t, $^3J_{H,H}$ = 6.9 Hz, 6H, CH_3).

2,2´-Di-*n*-octadecyloxy-4,4´´-bis[(2,2-dimethyl-1,3-dioxolane-4-yl)methoxy]-*p*-terphenyl 3/18: Synthesized from **2/18** (590 mg, 0.68 mmol), **1** (430 mg, 1.70 mmol), [$Pd(PPh_3)_4$] (50 mg, 0.03 mmol), purification by column chromatography (eluent: $CHCl_3$). Colorless solid, yield: 300 mg (43%), m. p. 43 °C, **^{1}H-NMR** ($CDCl_3$, 400 MHz): δ/ppm = 7.54 – 7.48 (m, 4H, Aryl-*H*), 7.08 (s, 2H, Aryl-*H*), 6.98 – 6.92 (m, 4H, Aryl-*H*), 4.51 (quin, $^3J_{H,H}$ = 5.97 Hz, 2H, OC*H*), 4.19 (dd, $^3J_{H,H}$ = 8.5, $^3J_{H,H}$ = 6.4 Hz, 2H, OCH_2), 4.16 – 4.08 (m, 2H, OCH_2), 4.04 – 3.90 (m, 4H, OCH_2), 3.76 (t, $^3J_{H,H}$ = 6.5 Hz, 4H, OCH_2), 1.58 – 1.50 (m, 4H, CH_2), 1.48 (s, 6H, $OC(CH_3)_2$), 1.42 (s, 6H, $OC(CH_3)_2$), 1.35 – 1.13 (m, 60H, CH_2), 0.88 (t, $^3J_{H,H}$ = 6.9 Hz, 6H, CH_3).

2,2-Di´-*n*-eicosyloxy-4,4´´-bis[(2,2-dimethyl-1,3-dioxolane-4-yl)methoxy]-*p*-terphenyl 3/20: Synthesized from **2/30** (230 mg, 0.25 mmol), **1** (160 mg, 0.62 mmol), [$Pd(PPh_3)_4$] (20 mg, 0.01 mmol), purification by column chromatography (eluent: $CHCl_3$). Colorless solid, yield: 220 mg (81%), m. p. 49 °C, **^{1}H-NMR** ($CDCl_3$, 400 MHz): δ / ppm = 7.53 – 7.48 (m, 4H, Aryl-*H*), 7.08 (s, 2H, Aryl-*H*), 6.98 – 6.93 (m, 4H, Aryl-*H*), 4.51 (quin, $^3J_{H,H}$ = 6.11 Hz, 2H, OC*H*), 4.19 (dd, $^3J_{H,H}$ = 8.4, $^3J_{H,H}$ = 6.4 Hz, 2H, OCH_2), 4.15 – 4.08 (m, 2H, OCH_2), 4.02 – 3.88 (m, 4H, OCH_2), 3.76 (t, $^3J_{H,H}$ = 6.6 Hz, 4H, OCH_2), 1.58 – 1.49 (m, 4H, CH_2), 1.48 (s, 6H, $OC(CH_3)_2$), 1.42 (s, 6H, $OC(CH_3)_2$), 1.36 – 1.13 (m, 68H, CH_2), 0.88 (t, $^3J_{H,H}$ = 6.9 Hz, 6H, CH_3).

2,2´-Di-*n*-docosyloxy-4,4´´-bis[(2,2-dimethyl-1,3-dioxolane-4-yl)methoxy]-*p*-terphenyl 3/22: Synthesized from **2/22** (560 mg, 0.57 mmol), **1** (320 mg, 1.25 mmol), [Pd(PPh_3)$_4$] (40 mg, 0.03 mmol), purification by column chromatography (eluent: $CHCl_3$). Colorless solid, yield: 300 mg (47%), m. p. 51 °C, **^{1}H-NMR** ($CDCl_3$, 400 MHz): δ / ppm = 7.53 – 7.48 (m, 4H, Aryl-*H*), 7.08 (s, 2H, Aryl-*H*), 6.98 – 6.93 (m, 4H, Aryl-*H*), 4.51 (quin, $^3J_{H,H}$ = 6.11 Hz, 2H, OC*H*), 4.19 (dd, $^3J_{H,H}$ = 8.4, $^3J_{H,H}$ = 6.4 Hz, 2H, OCH_2), 4.15 – 4.08 (m, 2H, OCH_2), 4.02 – 3.88 (m, 4H, OCH_2), 3.76 (t, $^3J_{H,H}$ = 6.6 Hz, 4H, OCH_2), 1.58 – 1.49 (m, 4H, CH_2), 1.48 (s, 6H, OC(CH_3)$_2$), 1.42 (s, 6H, OC(CH_3)$_2$), 1.36 – 1.13 (m, 76H, CH_2), 0.88 (t, $^3J_{H,H}$ = 6.9 Hz, 6H, CH_3).

2,2´-Di-*n*-tetracosyloxy-4,4´´-bis[(2,2-dimethyl-1,3-dioxolane-4-yl)methoxy]-*p*-terphenyl 2/24: Synthesized from **2/24** (310 mg, 0.30 mmol), **1** (200 mg, 0.66 mmol), [Pd(PPh_3)$_4$] (25 mg, 0.02 mmol), purification by column chromatography (eluent: $CHCl_3$). Colorless solid, yield: 210 mg (58%), m. p. 78 °C, **^{1}H-NMR** ($CDCl_3$, 400 MHz): δ / ppm = 7.53 – 7.48 (m, 4H, Aryl-*H*), 7.08 (s, 2H, Aryl-*H*), 6.98 – 6.93 (m, 4H, Aryl-*H*), 4.51 (quin, $^3J_{H,H}$ = 6.11 Hz, 2H, OC*H*), 4.19 (dd, $^3J_{H,H}$ = 8.4, $^3J_{H,H}$ = 6.4 Hz, 2H, OCH_2), 4.15 – 4.08 (m, 2H, OCH_2), 4.02 – 3.88 (m, 4H, OCH_2), 3.76 (t, $^3J_{H,H}$ = 6.6 Hz, 4H, OCH_2), 1.58 – 1.49 (m, 4H, CH_2), 1.48 (s, 6H, OC(CH_3)$_2$), 1.42 (s, 6H, OC(CH_3)$_2$), 1.36 – 1.13 (m, 84H, CH_2), 0.88 (t, $^3J_{H,H}$ = 6.9 Hz, 6H, CH_3).

2,2´-Di-*n*-hexacosyloxy-4,4´´-bis[(2,2-dimethyl-1,3-dioxolane-4-yl)methoxy]-*p*-terphenyl 2/26: Synthesized from **2/26** (250 mg, 0.23 mmol), **1** (130 mg, 0.51 mmol), [Pd(PPh_3)$_4$] (15 mg, 0.01 mmol), purification by column chromatography (eluent: $CHCl_3$). Colorless solid, yield: 250 mg (85%), m. p. 83 °C, **^{1}H-NMR** ($CDCl_3$, 400 MHz): δ / ppm = 7.53 – 7.48 (m, 4H, Aryl-*H*), 7.08 (s, 2H, Aryl-*H*), 6.98 – 6.93 (m, 4H, Aryl-*H*), 4.51 (quin, $^3J_{H,H}$ = 6.11 Hz, 2H, -OC*H*), 4.19 (dd, $^3J_{H,H}$ = 8.4, $^3J_{H,H}$ = 6.4 Hz, 2H, -OCH_2), 4.15 – 4.08 (m, 2H, -OCH_2), 4.02 – 3.88 (m, 4H, -OCH_2), 3.76 (t, $^3J_{H,H}$ = 6.6 Hz, 4H, -OCH_2), 1.58 – 1.49 (m, 4H, -CH_2), 1.48 (s, 6H, -OC(CH_3)$_2$), 1.42 (s, 6H, -OC(CH_3)$_2$), 1.36 – 1.13 (m, 92H, -CH_2), 0.88 (t, $^3J_{H,H}$ = 6.9 Hz, 6H, -CH_3).

2,2´-Di-*n*-triacontyloxy-4,4´´-bis[(2,2-dimethyl-1,3-dioxolane-4-yl)methoxy]-*p*-terphenyl 3/30: Synthesized from **2/30** (500 mg, 0.42 mmol), **1** (250 mg, 0.91 mmol), [Pd(PPh_3)$_4$] (30 mg, 0.02 mmol), purification by column chromatography (eluent: $CHCl_3$). Colorless solid, yield: 350 mg (61%), m. p. 85 °C, **^{1}H-NMR** ($CDCl_3$, 400 MHz): δ / ppm = 7.53 – 7.48 (m, 4H, Aryl-*H*), 7.08 (s, 2H, Aryl-*H*), 6.98 – 6.93 (m, 4H, Aryl-*H*), 4.51 (quin, $^3J_{H,H}$ = 6.11 Hz, 2H, -OC*H*), 4.19 (dd, $^3J_{H,H}$ = 8.4, $^3J_{H,H}$ = 6.4 Hz, 2H, -OCH_2), 4.15 – 4.08 (m, 2H, -OCH_2), 4.02 – 3.88 (m, 4H, -OCH_2), 3.76 (t, $^3J_{H,H}$ = 6.6 Hz, 4H, -OCH_2), 1.58 – 1.49 (m, 4H, -CH_2), 1.48 (s, 6H, -OC(CH_3)$_2$), 1.42 (s, 6H, -OC(CH_3)$_2$), 1.36 – 1.13 (m, 112H, -CH_2), 0.88 (t, $^3J_{H,H}$ = 6.9 Hz, 6H, -CH_3).

2,2´-Di-*n*-didotriacontyloxy-4,4´´-bis[(2,2-dimethyl-1,3-dioxolane-4-yl)methoxy]-*p*-terphenyl 3/32: Synthesized from **2/32** (300 mg, 0.24 mmol), **1** (160 mg, 0.62 mmol), [Pd(PPh_3)$_4$] (15 mg, 0.01 mmol), purification by column chromatography (eluent: $CHCl_3$). Colorless solid, yield: 280 mg (82%), m. p. 83 °C, **^{1}H-NMR** ($CDCl_3$, 400 MHz): δ / ppm = 7.54 – 7.48 (m, 4H, Aryl-*H*), 7.08 (s, 2H, Aryl-*H*), 6.98 – 6.92 (m, 4H, Aryl-*H*), 4.55 – 4.47 (m, 2H, OC*H*), 4.19 (dd, $^3J_{H,H}$ = 8.5 Hz, $^3J_{H,H}$ = 6.4 Hz, 2H, OCH_2), 4.14 – 4.08 (m, 2H, OCH_2), 4.03 – 3.90 (m, 4H, OCH_2), 3.76 (t, $^3J_{H,H}$ = 6.5 Hz, 4H, OCH_2), 1.54 (m, 4H, CH_2), 1.48 (s, 6H, C(CH_3)$_2$), 1.42 (s, 6H, C(CH_3)$_2$), 1.22 (m, 116H, CH_2), 0.88 (t, $^3J_{H,H}$ = 6.8 Hz, 6H, CH_3).

S3.3 Compounds π/*n*

A mixture of appropriate bis-isopropylidene acetal **3/*n*** (~0.2-0.6 mmol) was dissolved in MeOH (20 mL) and THF (20 mL) and diluted hydrochloric acid (10%, 5 mL) was added. The solution was stirred at reflux for 6 hrs.[S14] After cooling the mixture was diluted with water (50 mL) extracted with EtOAc (3x 50 mL) and the organic solution was washed with saturated $NaHCO_3$-sol., water and brine (50 mL each). After drying over Na_2SO_4 the solvent was removed in vacuo (rotatory evaporator) and the residue was purified by column chromatography and crystallization.

2,2´-Di-*n*-hexyloxy-4,4´´-bis(2,3-dihydroxypropyloxy)-*p*-terphenyl (π/6): Synthesized from **3/6** (410 mg, 0.59 mmol); purification by column chromatography (eluent: $CHCl_3$/MeOH = 9/1) and recrystallized from MeOH/H_2O. Colorless solid, $C_{36}H_{50}O_8$, M = 610.78 g/mol, yield: 250 mg (70%), **^{1}H NMR** (pyridine-d$_5$, 400 MHz): δ/ppm = 7.80 (d, $^3J_{H,H}$ = 8.7 Hz, 4H, Aryl-H), 7.34 (s, 2H, Aryl-H), 7.28 (d, $^3J_{H,H}$ = 8.8 Hz, 4H, Aryl-H), 4.70 – 4.56 (m, 4H, OCH_2), 4.56 – 4.46 (m, 2H, OCH), 4.35 – 4.21 (m, 4H, OCH_2), 3.99 (t, $^3J_{H,H}$ = 6.5 Hz, 4H, OCH_2), 1.75 – 1.61 (m, 4H, CH_2), 1.41 – 1.08 (m, 16H, CH_2), 0.84 (t, $^3J_{H,H}$ = 7.1 Hz, 6H, CH_3). **^{13}C NMR** (pyridine-d$_5$, 126 MHz): δ/ppm = 157.42, 149.35, 129.51, 129.19, 124.04, 112.99 (Aryl-C), 71.93 (OCH_2), 69.76 (OCH), 69.27, 62.69 (OCH_2), 30.02, 28.81, 24.25, 21.12 (CH_2), 12.39 (CH_3). HRMS (m/z): $[M]^+Cl^-$ calcd. for $C_{36}H_{50}O_8Cl$, 645.3189; found 645.3166.

2,2´-Di-*n*-octyloxy-4,4´´-bis(2,3-dihydroxypropyloxy)-*p*-terphenyl (π/8): Synthesized from **3/8** (430 mg, 0.57 mmol); purification by column chromatography (eluent: $CHCl_3$/MeOH = 9/1) and recrystallized from MeOH/H_2O. Colorless solid, $C_{40}H_{58}O_8$, M = 666.88 g/mol, yield: 270 mg (71%), **^{1}H NMR** (pyridine-d$_5$, 400 MHz): δ/ppm = 7.82 (d, $^3J_{H,H}$ = 8.7 Hz, 4H, Aryl-H), 7.35 (s, 2H, Aryl-H), 7.28 (d, $^3J_{H,H}$ = 8.8 Hz, 4H, Aryl-H), 4.68 – 4.56 (m, 4H, OCH_2), 4.55 – 4.47 (m, 2H, OCH), 4.33 – 4.21 (m, 4H, OCH_2), 4.02 (t, $^3J_{H,H}$ = 6.5 Hz, 4H, OCH_2), 1.79 – 1.64 (m, 4H, CH_2), 1.42 – 1.30 (m, 4H, CH_2), 1.30 – 1.13 (m, 20H, CH_2), 0.88 (t, $^3J_{H,H}$ = 7.0 Hz, 6H, CH_3). **^{13}C NMR** (pyridine-d$_5$, 126 MHz): δ/ppm = 157.43, 149.37, 129.48, 129.19, 124.00, 113.00 (Aryl-C), 71.95 (OCH_2), 69.74 (OCH), 69.22, 62.64 (OCH_2), 30.25, 28.88, 27.87, 27.75, 24.62, 21.11 (CH_2), 12.47 (CH_3). HRMS (m/z): $[M]^+Cl^-$ calcd. for $C_{40}H_{58}O_8Cl$, 701.3815; found 701.3777.

2,2´-Di-*n*-nonyloxy-4,4´´-bis(2,3-dihydroxypropyloxy)-*p*-terphenyl (π/9): Synthesized from **3/9** (480 mg, 0.62 mmol); purification by column chromatography (eluent: $CHCl_3$/MeOH = 9/1) and recrystallized from MeOH/H_2O. Colorless solid, $C_{42}H_{62}O_8$, M = 694.94 g/mol, yield: 310 mg (72%), **^{1}H NMR** (pyridine-d$_5$, 400 MHz): δ/ppm = 7.83 (d, $^3J_{H,H}$ = 8.8 Hz, 4H, Aryl-H), 7.35 (s, 2H, Aryl-H), 7.29 (d, $^3J_{H,H}$ = 8.8 Hz, 4H, Aryl-H), 4.69 – 4.56 (m, 4H, OCH_2), 4.56 – 4.47 (m, 2H, OCH), 4.34 – 4.22 (m, 4H, OCH_2), 4.04 (t, $^3J_{H,H}$ = 6.5 Hz, 4H, OCH_2), 1.80 – 1.67 (m, 4H, CH_2), 1.46 – 1.34 (m, 4H, CH_2), 1.30 (m, 20H, CH_2), 0.89 (t, $^3J_{H,H}$ = 6.8 Hz, 6H, CH_3). **^{13}C NMR** (pyridine-d$_5$, 126 MHz): δ/ppm = 158.99, 150.93, 149.99, 131.03, 130.75, 125.56, 114.56 (Aryl-C), 73.53 (OCH_2), 71.29 (OCH), 70.78, 64.18 (OCH_2), 31.86, 30.46, 29.63, 29.50, 29.32, 26.20, 22.69 (CH_2), 14.04 (CH_3), analysis (calcd. for $C_{42}H_{62}O_8$): C(72.59, 72.62), H(8.99, 8.60).

2,2´-Di-*n*-decyloxy-4,4´´-bis(2,3-dihydroxypropyloxy)-*p*-terphenyl (π/10): Synthesized from **3/10** (210 mg, 0.29 mmol); purification by column chromatography (eluent: $CHCl_3$/MeOH = 9/1) and recrystallized from MeOH. Colorless solid, $C_{44}H_{66}O_8$, M = 722.99 g/mol, yield: 160 mg (77%), **^{1}H NMR** (pyridine-d$_5$, 400 MHz): δ/ppm = 7.82 (d, $^3J_{H,H}$ = 8.6 Hz, 4H, Aryl-H), 7.35 (s, 2H, Aryl-H), 7.29 (d, $^3J_{H,H}$ = 8.7 Hz, 4H, Aryl-H), 4.67 – 4.57 (m, 4H, OCH_2), 4.56 – 4.48 (m, 2H, OCH),

4.33 – 4.21 (m, 4H, OCH_2), 4.03 (t, $^3J_{H,H}$ = 6.5 Hz, 4H, OCH_2), 1.81 – 1.67 (m, 4H, CH_2), 1.46 – 1.32 (m, 4H, CH_2), 1.32 – 1.17 (m, 28H, CH_2), 0.89 (t, $^3J_{H,H}$ = 6.8 Hz, 6H, CH_3). **^{13}C NMR** (pyridine-d_5, 126 MHz): δ/ppm = 159.00, 150.94, 131.04, 130.75, 125.57, 114.57 (Aryl-C), 73.53 (OCH_2), 71.31 (OCH), 70.79, 64.20 (OCH_2), 31.90, 30.47, 29.71, 29.64, 29.53, 29.38, 26.22, 22.71 (CH_2), 14.05 (CH_3). HRMS (m/z): $[M]^+Cl^-$ calcd. for $C_{44}H_{66}O_8Cl$, 757.4441; found 757.4401.

2,2′-Di-*n*-dodecyloxy-4,4′′-bis(2,3-dihydroxypropyloxy)-*p*-terphenyl (π/12): Synthesized from **3/12** (220 mg, 0.28 mmol); purification by column chromatography (eluent: $CHCl_3$/MeOH = 9/1) and recrystallized from MeOH. Colorless solid, $C_{48}H_{74}O_8$, M = 779.10 g/mol, yield: 150 mg (70%), **^{1}H NMR** (pyridine-d_5, 400 MHz): δ/ppm = 7.83 (d, $^3J_{H,H}$ = 8.8 Hz, 4H, Aryl-H), 7.35 (s, 2H, Aryl-H), 7.29 (d, $^3J_{H,H}$ = 8.8 Hz, 4H, Aryl-H), 4.69 – 4.56 (m, 4H, OCH_2), 4.56 – 4.47 (m, 2H, OCH), 4.34 – 4.22 (m, 4H, OCH_2), 4.04 (t, $^3J_{H,H}$ = 6.5 Hz, 4H, OCH_2), 1.80 – 1.67 (m, 4H, CH_2), 1.46 – 1.34 (m, 4H, CH_2), 1.30 (m, 36H, CH_2), 0.89 (t, $^3J_{H,H}$ = 6.8 Hz, 6H, CH_3). **^{13}C NMR** (pyridine-d_5, 126 MHz): δ/ppm = 159.00, 150.95, 131.05, 130.75, 125.58, 114.58 (Aryl-C), 73.53 (OCH_2), 71.30 (OCH), 70.79, 64.19 (OCH_2), 31.92, 30.49, 29.76, 29.75, 29.73, 29.56, 29.41, 26.24, 22.72 (CH_2), 14.05 (CH_3). HRMS (m/z): $[M]^+Cl^-$ calcd. for $C_{48}H_{74}O_8Cl$, 813.5067; found 813.5051.

2,2′-Di-*n*-tetradecyloxy-4,4′′-bis(2,3-dihydroxypropyloxy)-*p*-terphenyl (π/14): Synthesized from **3/14** (300 mg, 0.28 mmol); purification by column chromatography (eluent: $CHCl_3$/MeOH = 9/1) and recrystallized from MeOH. Colorless solid, $C_{52}H_{82}O_8$, M = 835.20 g/mol, yield: 150 mg (55%), **^{1}H NMR** (pyridine-d_5, 400 MHz): δ/ppm = 7.83 (d, $^3J_{H,H}$ = 8.7 Hz, 4H, Aryl-H), 7.35 (s, 2H, Aryl-H), 7.29 (d, $^3J_{H,H}$ = 8.8 Hz, 4H, Aryl-H), 4.63 (m, 4H, OCH_2), 4.52 (m, 2H, OCH), 4.33 – 4.22 (m, 4H, OCH_2), 4.04 (t, $^3J_{H,H}$ = 6.5 Hz, 4H, OCH_2), 1.82 – 1.69 (m, 4H, CH_2), 1.47 – 1.35 (m, 4H, CH_2), 1.35 – 1.19 (m, 44H, CH_2), 0.89 (t, $^3J_{H,H}$ = 6.8 Hz, 6H, CH_3). **^{13}C NMR** (pyridine-d_5, 101 MHz): δ/ppm = 157.80, 149.74, 129.84, 129.55, 124.38, 113.38 (Aryl-C), 72.33 (OCH_2), 70.10 (OCH), 69.59, 62.99 (OCH_2), 30.71, 29.29, 28.60, 28.59, 28.55, 28.53, 28.37, 28.21, 25.05, 21.51 (CH_2), 12.85 (CH_3). HRMS (m/z): $[M]^+Cl^-$ calcd. for $C_{52}H_{82}O_8Cl$, 869.5693; found 869.5684.

2,2′-Di-*n*-hexadecyloxy-4,4′′-bis(2,3-dihydroxypropyloxy)-*p*-terphenyl (π/16): Synthesized from **3/16** (390 mg, 0.40 mmol); purification by column chromatography (eluent: $CHCl_3$/MeOH = 9/1) and recrystallized from MeOH. Colorless solid, $C_{56}H_{90}O_8$, M = 891.31 g/mol, yield: 190 mg (54%), **^{1}H NMR** (pyridine-d_5, 400 MHz): δ/ppm = 7.83 (d, $^3J_{H,H}$ = 8.7 Hz, 4H, Aryl-H), 7.35 (s, 2H, Aryl-H), 7.29 (d, $^3J_{H,H}$ = 8.8 Hz, 4H, Aryl-H), 4.68 – 4.57 (m, 4H, OCH_2), 4.52 (m, 2H, OCH), 4.33 – 4.22 (m, 4H, OCH_2), 4.04 (t, $^3J_{H,H}$ = 6.5 Hz, 4H, OCH_2), 1.74 (m, 4H, CH_2), 1.41 (m, 4H, CH_2), 1.31 (m, 52H, CH_2), 0.89 (t, $^3J_{H,H}$ = 6.8 Hz, 6H, CH_3). **^{13}C NMR** (pyridine-d_5, 101 MHz): δ/ppm = 157.80, 149.75, 129.84, 129.55, 124.38, 113.38 (Aryl-C), 72.33 (OCH_2), 70.09 (OCH), 69.59, 62.99 (OCH_2), 30.70, 29.29, 28.62, 28.62, 28.60, 28.58, 28.57, 28.51, 28.37, 28.20, 25.05, 21.51 (CH_2), 12.85 (CH_3). HRMS (m/z): $[M]^+Cl^-$ calcd. for $C_{56}H_{90}O_8Cl$, 925.6319; found 925.6300.

2,2′-Di-*n*-octadecyloxy-4,4′′-bis(2,3-dihydroxypropyloxy)-*p*-terphenyl (π/18): Synthesized from **3/18** (300 mg, 0.40 mmol); purification by column chromatography (eluent: $CHCl_3$/MeOH = 9/1) and recrystallized from MeOH. Colorless solid, $C_{60}H_{98}O_8$, M = 947.42 g/mol, yield: 250 mg (90%), **^{1}H NMR** (pyridine-d_5, 400 MHz): δ/ppm 7.83 (d, $^3J_{H,H}$ = 8.7 Hz, 4H, Aryl-*H*), 7.35 (s, 2H, Aryl-*H*), 7.29 (d, $^3J_{H,H}$ = 8.7 Hz, 4H, Aryl-*H*), 4.62 (m, 4H, OC*H*2), 4.52 (m, 2H, OC*H*), 4.34 – 4.22 (m, 4H, OC*H*2), 4.04 (t, $^3J_{H,H}$ = 6.5 Hz, 4H, OC*H*2), 1.81 – 1.68 (m, 4H, C*H*2), 1.49 – 1.36 (m,

4H, CH_2), 1.32 (m, 60H, CH_2), 0.89 (t, $^3J_{H,H}$ = 6.8 Hz, 6H, CH_3). **^{13}C NMR** (pyridine-d$_5$, 101 MHz): δ/ppm = 157.80, 149.75, 129.84, 129.55, 124.38, 113.38 (Aryl-*C*), 72.34 (OCH$_2$), 70.10 (OCH), 69.59, 63.00 (O*C*H$_2$), 30.70, 29.29, 28.62, 28.59, 28.57, 28.51, 28.38, 28.19, 25.06, 21.51 (*C*H$_2$), 12.85 (*C*H$_3$). Analysis (calcd. for $C_{60}H_{98}O_8$): C (75.95, 75.40), H (9.30, 10.11). HRMS (m/z): $[M]^+Cl^-$ calcd. for $C_{60}H_{98}O_8Cl$, 981.6945; found 981.6895.

2,2´-Di-*n*-eicosyloxy-4,4´´-bis(2,3-dihydroxypropyloxy)-*p*-terphenyl (π/20): Synthesized from **3/20** (220 mg, 0.40 mmol); purification by column chromatography (eluent: $CHCl_3$/MeOH = 9/1) and recrystallized from MeOH. Colorless solid, $C_{64}H_{106}O_8$, M = 1003.52 g/mol, yield: 210 mg (95%), **^{1}H NMR** (pyridine-d$_5$, 400 MHz): δ/ppm = 7.83 (d, $^3J_{H,H}$ = 8.7 Hz, 4H, Aryl-H), 7.35 (s, 2H, Aryl-H), 7.29 (d, $^3J_{H,H}$ = 8.9 Hz, 4H, Aryl-H), 4.69 – 4.57 (m, 4H, OCH$_2$), 4.52 (dd, $^3J_{H,H}$ = 9.1, $^3J_{H,H}$ = 5.9 Hz, 2H, OCH), 4.33 – 4.22 (m, 4H, OCH$_2$), 4.04 (t, $^3J_{H,H}$ = 6.4 Hz, 4H, OCH$_2$), 1.82 – 1.69 (m, 4H, CH$_2$), 1.45 – 1.37 (m, 4H, CH$_2$), 1.37 – 1.20 (m, 68H, CH$_2$), 0.89 (t, $^3J_{H,H}$ = 6.8 Hz, 6H, CH$_3$). **^{13}C NMR** (pyridine-d$_5$, 101 MHz): δ/ppm = 157.81, 148.99, 129.84, 129.55, 124.38, 113.38 (Aryl-C), 72.34 (OCH$_2$), 70.10 (OCH), 69.59, 62.99 (OCH$_2$), 30.70, 29.30, 28.64, 28.62, 28.61, 28.59, 28.58, 28.56, 28.50, 28.38, 28.19, 25.06, 21.51 (CH$_2$), 12.85 (CH$_3$). HRMS (m/z): $[M]^+Cl^-$ calcd. for $C_{64}H_{106}O_8Cl$, 1037.7571; found 1037.7499.

2,2´-Di-*n*-docosyloxy-4,4´´-bis(2,3-dihydroxypropyloxy)-*p*-terphenyl (π/22): Synthesized from **3/22** (300 mg, 0.26 mmol); purification by column chromatography (eluent: $CHCl_3$/MeOH = 9/1) and recrystallized from MeOH/THF. Colorless solid, $C_{68}H_{114}O_8$, M = 1059.63 g/mol, yield: 270 mg (95%), **^{1}H NMR** (pyridine-d$_5$, 400 MHz): δ/ppm = 7.83 (d, $^3J_{H,H}$ = 8.8 Hz, 4H, Aryl-H), 7.35 (s, 2H, Aryl-H), 7.29 (d, $^3J_{H,H}$ = 8.7 Hz, 4H, Aryl-H), 6.90 (s, 2H, OH), 6.50 (s, 2H, OH), 4.71 – 4.56 (m, 4H, OCH$_2$), 4.52 (dd, $^3J_{H,H}$ = 9.2, $^3J_{H,H}$ = 5.9 Hz, 2H, OCH), 4.28 (m, 4H, OCH$_2$), 4.04 (t, $^3J_{H,H}$ = 6.5 Hz, 4H, OCH$_2$), 1.82 – 1.67 (m, 4H, CH$_2$), 1.51 – 1.17 (m, 76H, CH$_2$), 0.89 (t, $^3J_{H,H}$ = 6.8 Hz, 6H, CH$_3$). **^{13}C NMR** (pyridine-d$_5$, 101 MHz): δ/ppm = 157.80, 149.75, 129.84, 129.55, 124.38, 113.38 (Aryl-C), 72.34 (OCH$_2$), 70.11 (OCH), 69.59, 63.00 (OCH$_2$), 30.70, 29.30, 28.64, 28.63, 28.61, 28.58, 28.56, 28.50, 28.39, 28.19, 25.06, 21.51 (CH$_2$), 12.85 (CH$_3$). HRMS (m/z): $[M]^+Cl^-$ calcd. for $C_{68}H_{114}O_8Cl$, 1093.8197; found 1093.8157.

2,2´-Di-*n*-tetracosyloxy-4,4´´-bis(2,3-dihydroxypropyloxy)-*p*-terphenyl (π/24): Synthesized from **3/24** (210 mg, 0.18 mmol); purification by column chromatography (eluent: $CHCl_3$/MeOH = 9/1) and recrystallized from EtOAc. Colorless solid, $C_{72}H_{122}O_8$, M = 1115.73 g/mol, yield: 130 mg (66%), **^{1}H NMR** (pyridine-d$_5$, 500 MHz): δ/ppm = 7.81 (d, $^3J_{H,H}$ = 8.8 Hz, 4H, Aryl-H), 7.33 (s, 2H, Aryl-H), 7.28 (d, $^3J_{H,H}$ = 8.8 Hz, 4H, Aryl-H), 4.67 – 4.55 (m, 4H, OCH$_2$), 4.53 – 4.46 (m, 2H, OCH), 4.30 – 4.21 (m, 4H, OCH$_2$), 4.02 (t, $^3J_{H,H}$ = 6.4 Hz, 4H, OCH$_2$), 1.79 – 1.68 (m, 4H, CH$_2$), 1.47 – 1.36 (m, 4H, CH$_2$), 1.36 – 1.18 (m, 80H, CH$_2$), 0.87 (t, $^3J_{H,H}$ = 7.0 Hz, 6H, CH$_3$). **^{13}C NMR** (pyridine-d$_5$, 126 MHz): δ/ppm = 159.00, 150.95, 131.05, 130.75, 125.58, 114.59 (Aryl-C), 73.54 (OCH$_2$), 71.31 (OCH), 70.79, 64.20 (OCH$_2$), 31.90, 30.50, 29.85, 29.83, 29.82, 29.81, 29.78, 29.77, 29.76, 29.70, 29.59, 29.39, 26.27, 22.71 (CH$_2$), 14.05 (CH$_3$). HRMS (m/z): $[M]^+Cl^-$ calcd. for $C_{72}H_{122}O_8Cl$, 1149.8823; found 1149.8743.

2,2´-Di-*n*-hexacosyloxy-4,4´´-bis(2,3-dihydroxypropyloxy)-*p*-terphenyl (π/26): Synthesized from **3/26** (250 mg, 0.20 mmol); purification by column chromatography (eluent: $CHCl_3$/MeOH = 9/1) and recrystallized from EtOAc. Colorless solid, $C_{76}H_{130}O_8$, M = 1171.84 g/mol, yield: 120 mg (52%), **^{1}H NMR** (pyridine-d$_5$, 400 MHz): δ/ppm = 7.83 (d, $^3J_{H,H}$ = 8.8 Hz, 4H, Aryl-H), 7.35 (s, 2H, Aryl-H), 7.29 (d, $^3J_{H,H}$ = 8.8 Hz, 4H, Aryl-H), 4.70 – 4.56 (m, 4H, OCH$_2$), 4.56 – 4.47 (m, 2H,

OCH), 4.35 – 4.22 (m, 4H, OCH_2), 4.04 (t, $^3J_{H,H}$ = 6.4 Hz, 4H, OCH_2), 1.84 – 1.66 (m, 4H, CH_2), 1.49 – 1.38 (m, 4H, CH_2), 1.38 – 1.21 (m, 88H, CH_2), 0.89 (t, $^3J_{H,H}$ = 6.9 Hz, 6H, CH_3). **^{13}C NMR** (pyridine-d_5, 101 MHz): δ/ppm = 158.99, 150.94, 131.03, 130.74, 125.57, 114.57 (Aryl-C), 73.53 (OCH_2), 71.28 (OCH), 70.77, 64.18 (OCH_2), 31.89, 30.49, 29.84, 29.83, 29.80, 29.78, 29.75, 29.69, 29.58, 29.38, 26.26, 22.70 (CH_2), 14.04 (CH_3). HRMS (m/z): $[M]^+Cl^-$ calcd. for $C_{76}H_{130}O_8Cl$, 1205.9449; found 1205.9381.

2,2′-Di-*n*-triacontyloxy-4,4′′-bis(2,3-dihydroxypropyloxy)-*p*-terphenyl (π/30): Synthesized from **3/30** (350 mg, 0.26 mmol); purification by column chromatography (eluent: $CHCl_3$/MeOH = 9/1) and recrystallized from EtOAc. Colorless solid, $C_{84}H_{146}O_8$, M = 1284.05 g/mol, yield: 170 mg (51%), **^{1}H NMR** (pyridine-d_5, 400 MHz): δ / ppm = 7.80 (d, J = 8.7 Hz, 4H, Ar-H), 7.32 (s, 2H, Ar-H), 7.26 (d, J = 8.7 Hz, 4H, Ar-H), 6.88 (s, br., 2H, OH), 6.47 (s, br, 2H, OH), 4.67 – 4.53 (m, 4H), 4.53 – 4.44 (m, 2H), 4.31 – 4.18 (m, 4H, CH_2O), 4.01 (t, J = 6.5 Hz, 4H, CH_2O), 1.80 – 1.64 (m, 4H, CH_2), 1.49 – 1.12 (m, 108H, CH_2), 0.86 (t, J = 6.9 Hz, 6H, CH_3); **^{13}C NMR** (pyridine-d_5, 101 MHz): δ/ppm = 159.00, 150.94, 131.17, 130.69, 125.48, 114.64 (Aryl-C), 73.54 (OCH_2), 71.26 (OCH), 70.81, 64.16 (OCH_2), 31.84, 30.45, 29.77, 29.75, 29.73, 29.69 (multiple C), 29.67, 29.62, 29.52, 29.30, 29.20, 22. 52 (CH_2), 13.93 (CH_3); analysis (calcd. for $C_{84}H_{146}O_8$): C 78.77 (78.57), H 11.63 (11.46).

2,2′-Di-*n*-dotriacontyloxy-4,4′′-bis(2,3-dihydroxypropyloxy)-*p*-terphenyl (π/32): Synthesized from **3/32** (280 mg, 0.20 mmol); purification by recrystallization from THF/MeOH. Colorless solid, $C_{88}H_{154}O_8$, M = 1340.16 g/mol, yield: 120 mg (45%), **^{1}H NMR** (pyridine-d_5, 400 MHz): δ / ppm = 7.79 (d, J = 8.7 Hz, 4H, Ar-H), 7.32 (s, 2H, Ar-H), 7.26 (d, J = 8.7 Hz, 4H, Ar-H), 4.60 – 4.55 (m, 4H), 4.53 – 4.44 (m, 2H), 4.26 – 4.17 (m, 4H, CH_2O), 4.04 (t, J = 6.5 Hz, 4H, CH_2O), 1.80 – 1.70 (m, 4H, CH_2), 1.49 – 1.12 (m, 116H, CH_2), 0.90 (t, J = 6.9 Hz, 6H, CH_3), **^{13}C NMR** (pyridine-d_5, 101 MHz): δ/ppm = 160.52, 152.45, 132.71, 132.19, 126.96, 116.17 (Aryl-C), 75.06 (OCH_2), 72.76 (OCH), 72.33, 65.65 (OCH_2), 33.33, 31.95, 31.26, 31.22, 31.19 (multiple C), 31.17, 31.11, 31.02, 30.79, 27.70, 24.11, (CH_2), 15.40 (CH_3); analysis (calcd. for $C_{88}H_{154}O_8$): C 78.48 (78.87), H 11.31 (11.58).

S3.4 Representative NMR Spectra

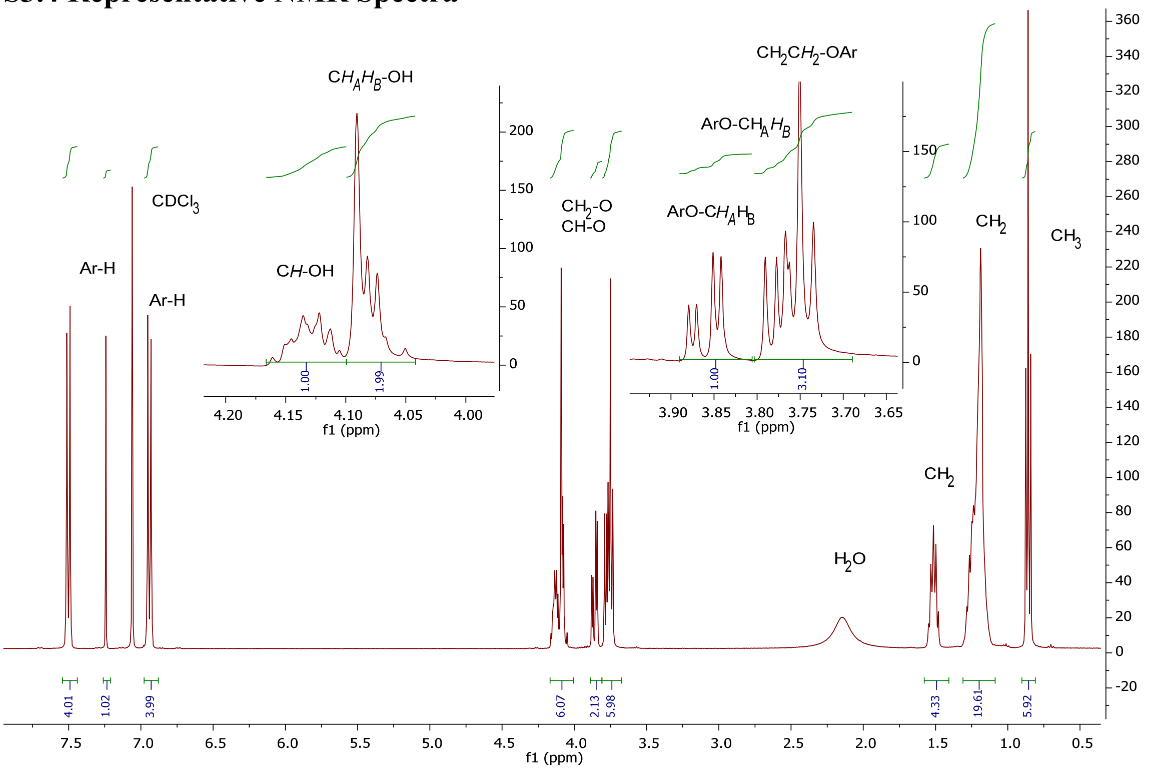

Figure S8a. ^{1}H-NMR spectrum of compound **π/8** (400 MHz, $CDCl_3$, 27 °C).

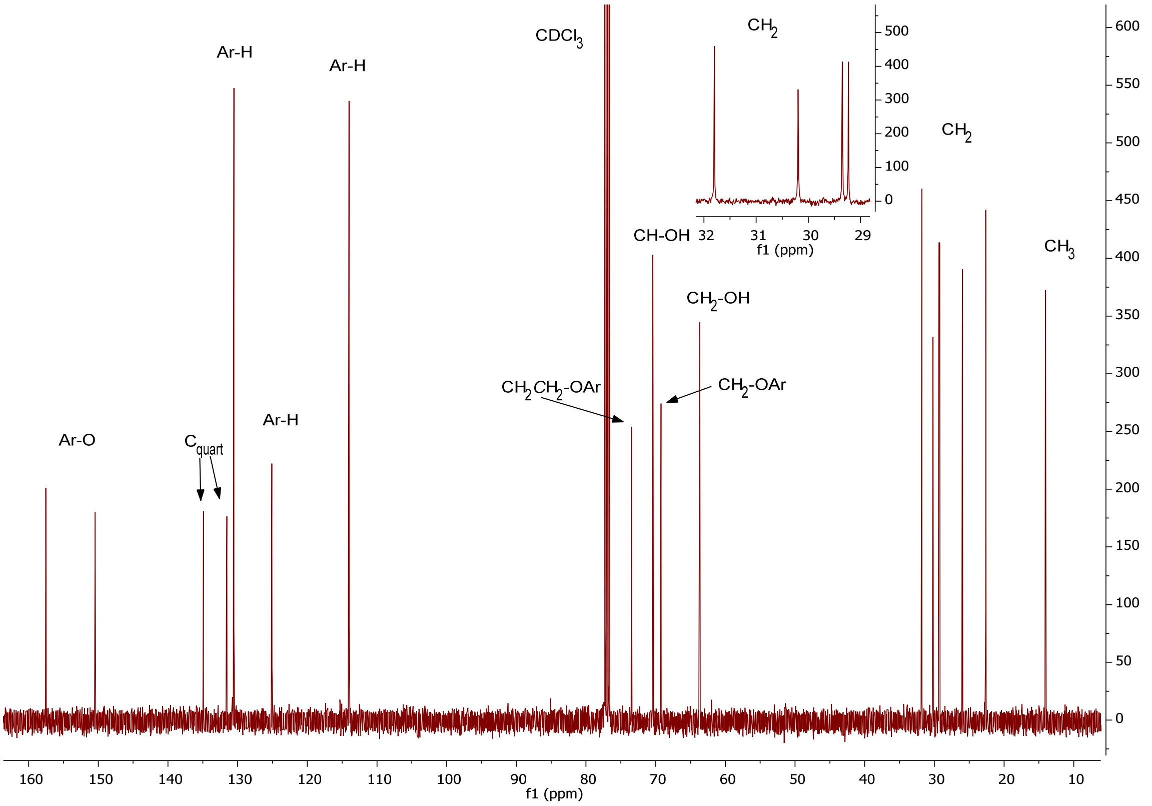

Figure S8b. ^{13}C-NMR spectrum of compound **π/8** (101 MHz, $CDCl_3$, 27 °C).

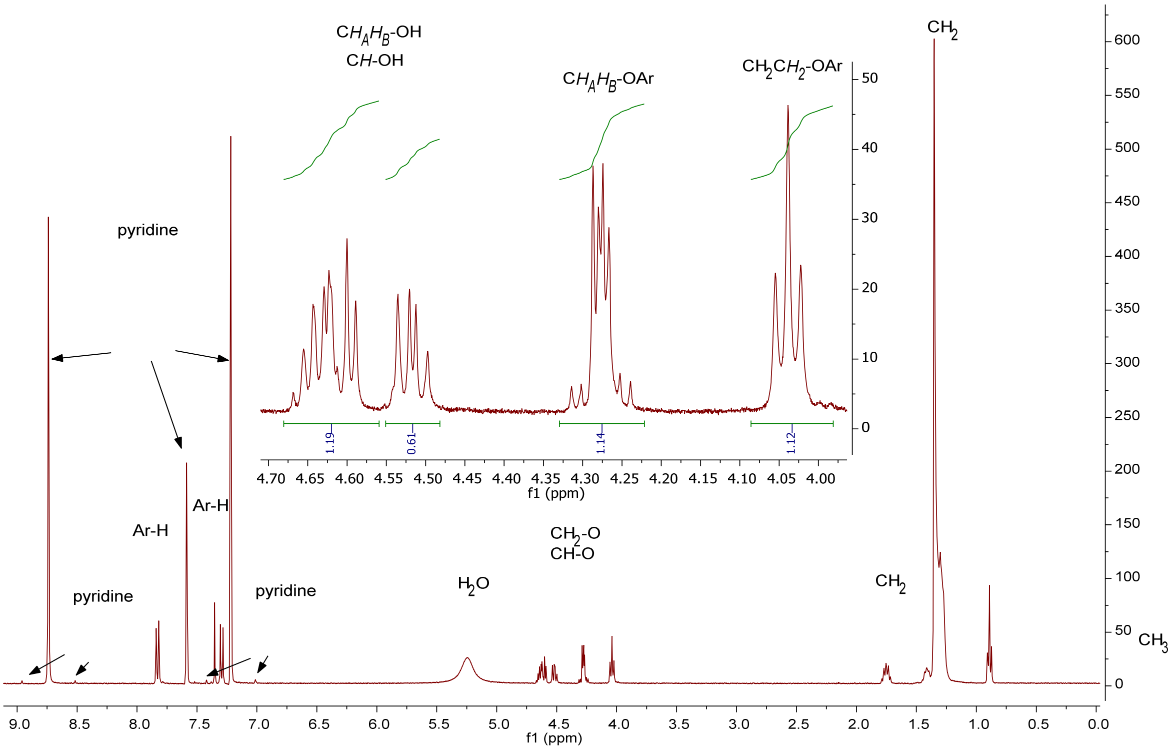

Figure S9a. ^{1}H-NMR spectrum of compound **π/26** (400 MHz, pyridine-d5, 27 °C).

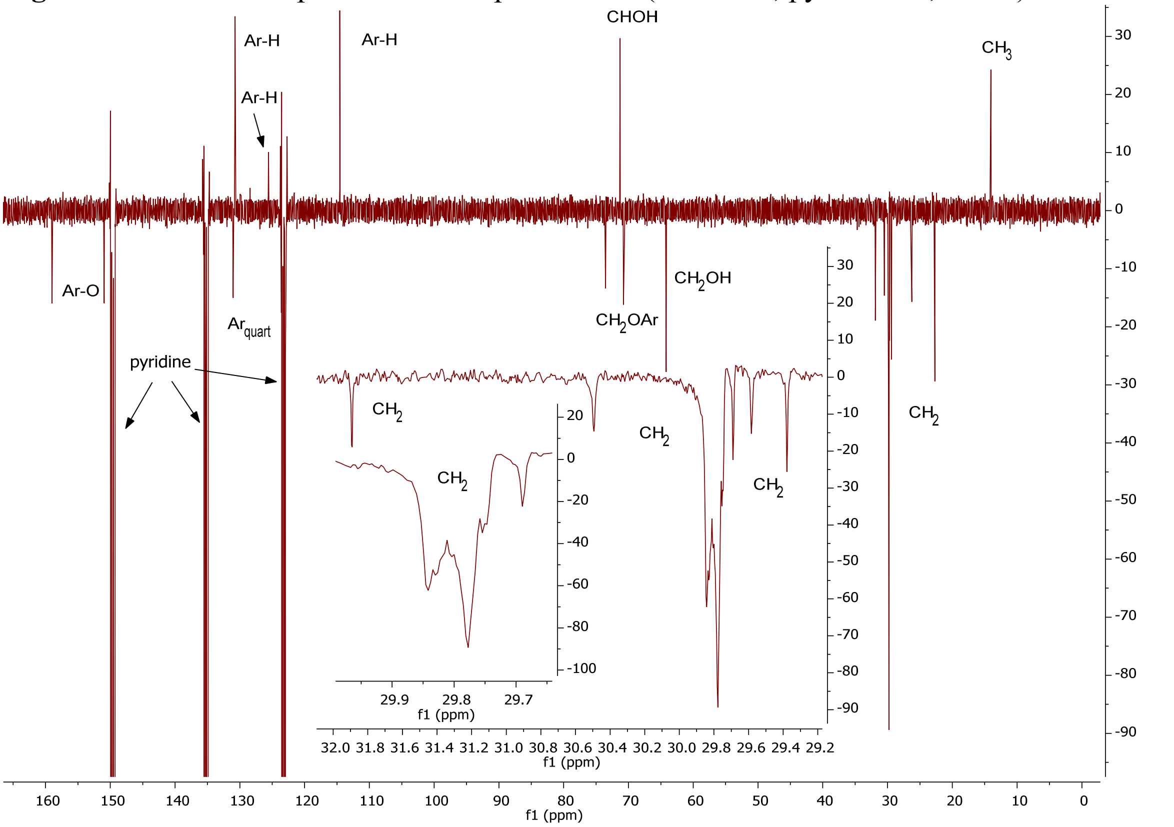

Figure S9b. ^{13}C-APT-NMR spectrum of compound **π/26** (101 MHz, pyridine-d5, 27 °C). One C_{ar} is hidden under the pyridine signal around 135 ppm.

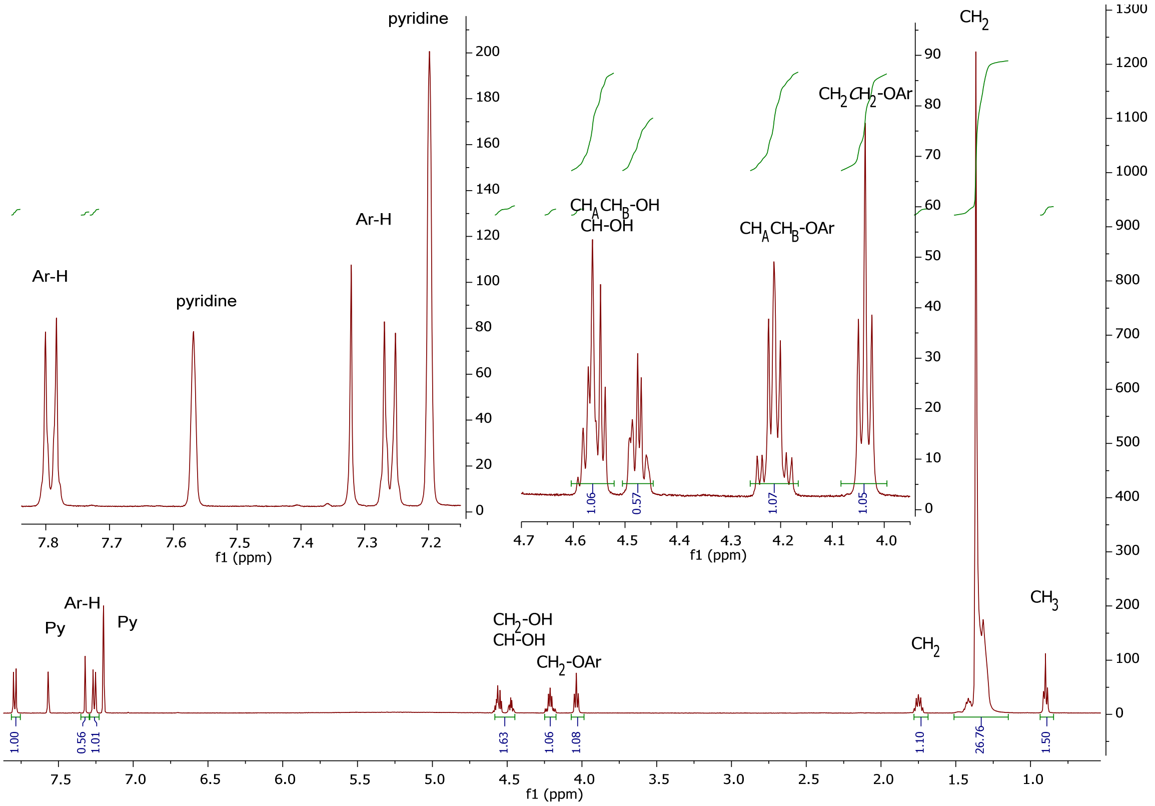

Figure S10a. ^{1}H-NMR spectrum of compound **π/32** (400 MHz, pyridine-d$_5$, 27 °C).

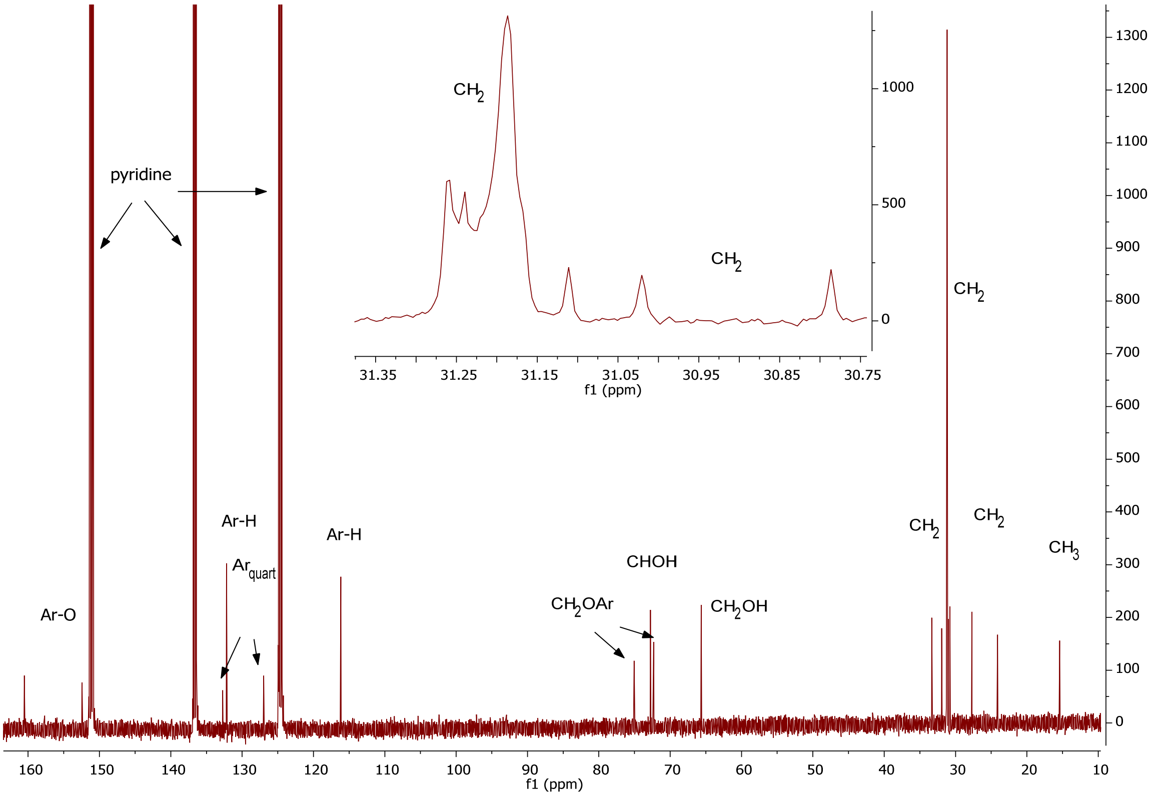

Figure S10b. ^{13}C-NMR spectrum of compound **π/32** (101 MHz, pyridine-d$_5$, 27 °C).

S4. References and Footnotes

[S1] A. Immirzi, B. Perini, *Acta Cryst*. **1977**, *33*, 216-218.

[S2] The factor 0.89 is a correction for the packing density of the LC state related to the crystalline, assuming an average of the packing density of between the liquid (0.55) and in the crystalline state (0.70). The calculated packing density would be (0.55+0.70)/2 = 0.625, corresponding to 0.89 times the packing density in the crystalline state (0,625/0.70 = 0.89).

[S3] C. Anders, V.-M. Fischer, T. Tan, M. Alaasar, R. Waldecker, Y. Ke, Y. Cao, F. Liu, C. Tschierske, *J. Mater. Chem*. **2025**, 13, 37-53.

[S4] S. Poppe, A. Lehmann, M. Steimecke, M. Prehm, Y. Zhao, C. Chen, Y, Cao, F. Liu, C. Tschierske, *Giant,* **2024**, *18*, 100254.

[S5] A. Lehmann, A. Scholte, M. Prehm, F. Liu, X. Zeng, G. Ungar, C. Tschierske, *Adv. Funct. Mater*. **2018**, 1804162.

[S6] Z. Zhu, T. M. Swager, *Org. Lett*. **2001**, *3*, 3471–3474.

[S7] M. Kölbel, T. Beyersdorff, X. Cheng, C. Tschierske, J. Kain, S. Diele,. *J. Am. Chem. Soc.* **2001**, *123*, 6809-6818.

[S8] J. R. Al Dulayymi, M. S. Baird, E. Roberts, *Tetrahedron* **2005**, *61*, 11939–11951.

[S9] A. Scholte, S. Hauche, M. Wagner, M. Prehm, S. Poppe, C. Chen, F. Liu, X. Zeng, G. Ungar, C. Tschierske, *Chem. Commun*. **2020**, *56*, 62-65.

[S10] S. Poppe, M. Poppe, H. Ebert, M. Prehm, C. Chen, F. Liu, S. Werner, K. Bacia, C. Tschierske, *Polymers* **2017**, *9*, 471.

[S11] H. Shimizu, K. Fujimoto, M. Furusyo, H. Maeda, Y. Nanai, K. Mizuno, M. Inouye, *J. Org. Chem*. **2007**, *72*, 1530-1533.

[S12] B. H. Northrop, A. Glöckner, P. J. Stang, *J. Org. Chem*. **2008**, *73*, 1787-1794.

[S13] N. Miyaura, T. Yanagi, A. Suzuki, *Synth. Commun*. **1981**, *11*, 513-519.

[S14] K. Heyns, J. Weyer, H. Paulsen, *Chem. Ber*. **1967**, *100*, 2317-2334.
